# Argonaute binding within human nuclear RNA and its impact on alternative splicing

**DOI:** 10.1261/rna.078707.121

**Published:** 2021-09

**Authors:** Yongjun Chu, Shinnichi Yokota, Jing Liu, Audrius Kilikevicius, Krystal C. Johnson, David R. Corey

**Affiliations:** UT Southwestern Medical Center, Departments of Pharmacology and Biochemistry, Dallas, Texas 75205, USA

**Keywords:** RNA, RNA interference, RNAi, micro RNA, miRNA, Argonaute

## Abstract

Mammalian RNA interference (RNAi) is often linked to the regulation of gene expression in the cytoplasm. Synthetic RNAs, however, can also act through the RNAi pathway to regulate transcription and splicing. While nuclear regulation by synthetic RNAs can be robust, a critical unanswered question is whether endogenous functions for nuclear RNAi exist in mammalian cells. Using enhanced crosslinking immunoprecipitation (eCLIP) in combination with RNA sequencing (RNA-seq) and multiple *AGO* knockout cell lines, we mapped AGO2 protein binding sites within nuclear RNA. The strongest AGO2 binding sites were mapped to micro RNAs (miRNAs). The most abundant miRNAs were distributed similarly between the cytoplasm and nucleus, providing no evidence for mechanisms that facilitate localization of miRNAs in one compartment versus the other. Beyond miRNAs, most statistically significant AGO2 binding was within introns. Splicing changes were confirmed by RT-PCR and recapitulated by synthetic miRNA mimics complementary to the sites of AGO2 binding. These data support the hypothesis that miRNAs can control gene splicing. While nuclear RNAi proteins have the potential to be natural regulatory mechanisms, careful study will be necessary to identify critical RNA drivers of normal physiology and disease.

## INTRODUCTION

The power of RNA interference (RNAi) to repress translation in the cytoplasm of mammalian cells is well known (Bartel 2018). Argonaute (AGO) proteins are the primary protein factors that facilitate RNAi (Meister 2013). There are four AGO proteins in human cells, AGO1−4. AGO2 is the best studied and the primary AGO variant capable of cleaving target RNAs (Liu et al. 2004; Meister et al. 2004). The roles of AGO1 and AGO3 are less clear, whereas AGO4 has been observed to make the least contribution to RNAi (Petri et al. 2011). Recently, AGO3 has been reported to be catalytically activated by shorter guide RNAs (Park et al. 2020).

The first step of endogenous regulation of physiologic processes in the cytoplasm involves the recognition of microRNAs (miRNAs) by AGO proteins. The miRNA:AGO complex is generally assumed to recognize sequences within the 3′-untranslated region (3′-UTR) of genes through complementary binding to a “seed sequence” at bases 2–8 of the miRNA, leading to repression of translation.

While the most attention has been focused on the impact of RNAi on translation (Zeng and Cullen 2002), miRNAs and RNAi protein factors (including AGO variants) also reside in cell nuclei (Robb et al. 2005; Jeffries et al. 2011; Gagnon et al. 2014a). The presence of key components of the RNAi machinery in cell nuclei suggests that miRNAs may also have the potential to control transcription, splicing, and other nuclear processes.

Evidence supporting the hypothesis that nuclear RNAi controls gene expression includes reports that small synthetic RNAs can be used to control transcription or splicing (Kalantari et al. 2016). Several laboratories have reported that promoter-target duplex RNAs can either repress or up-regulate transcription (Morris et al. 2004; Janowski et al. 2005, 2007; Li et al. 2006; Huang et al. 2010; Matsui et al. 2010, 2013; Zhang et al. 2014; Portnoy et al. 2016). Transcription can be controlled by synthetic miRNAs (Kim et al. 2008; Place et al. 2008; Younger and Corey 2011), and an endogenous miRNA can control endogenous cyclooxygenase 2 (COX-2) expression by binding to a transcript that overlaps the COX-2 promoter (Matsui et al. 2013).

Small RNAs can also regulate splicing (Allo et al. 2009; Liu et al. 2012). Small RNAs that target key regions near intron/exon boundaries have been shown to modulate splicing of an engineered luciferase model gene, dystrophin, and survival motor neuron 2 (SMN2) (Liu et al. 2012). This mechanism is like that of antisense oligonucleotides that target the same sites to affect alternative splicing by blocking binding of splicing factors (Havens and Hastings 2016). Small synthetic RNAs can also target intronic RNA to couple chromatin silencing and alternative splicing (Allo et al. 2009; Ameyar-Zazoua et al. 2012). These studies imply that RNA:AGO complexes may be acting as ribonucleoprotein splicing factors, combining the versatile recognition properties of RNA with the stabilizing and bridging properties of proteins.

While these data related to the nuclear activities of synthetic duplex RNAs are intriguing, there have been no reports of endogenous miRNAs controlling splicing. An outstanding question, therefore, is whether the robust and versatile control achieved using designed synthetic RNAs reflects an unappreciated layer of natural regulation of transcription and splicing by small RNAs. To begin to answer this question it is necessary to understand where miRNA recognition occurs in the nuclear transcriptome and what consequences that recognition has for gene expression.

Here we use enhanced crosslinking immunoprecipitation (eCLIP) (Van Nostrand et al. 2016) to determine AGO2 localization within the transcriptome of mammalian cell nuclei. We then examined the effects of knocking out *AGO1*, *AGO2*, *AGO1/2*, *AGO1/2/3*, and *DROSHA* on alternative splicing for genes with significant intronic AGO2-binding sites. Our results suggest that AGO2 protein binds to sites with introns and support the conclusion that alternative splicing can be achieved by miRNAs. TNRC6 proteins that bind AGO proteins and act in concert during RNAi exhibit a similar impact on alternative splicing (see Johnson et al. 2021).

## RESULTS

### Experimental design

We anticipated that it would be necessary to knock out more than one *AGO* variant. Therefore, we focused our analysis on HCT116 colorectal cancer-derived cells because they are diploid, facilitating the knockout of multiple genes. Another advantage is that miRNA expression in HCT116 cells is representative of miRNA expression as measured in a comprehensive study of approximately 1000 cancer cell lines (Ghandi et al. 2019), making it reasonable to expect that our data would be representative of many other widely used cultured cell lines. We also have *DROSHA* and *TNRC6* paralogs (see Johnson et al. 2021) knocked out in HCT116 cells, allowing direct comparison with other components of the RNAi pathway.

**FIGURE 1.**
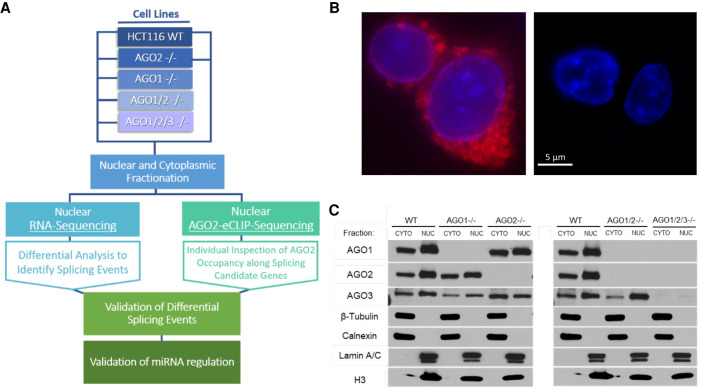
Experimental scheme for AGO2-eCLIP-sequencing. (*A*) Scheme showing how knockout of *AGO* variants, eCLIP, and nuclear RNA-seq analysis of alternative splicing relative to wild-type cells are used to identify and validate candidate genes. Wild-type and HCT116 knockout cells were used for RNA-seq. Wild-type and AGO2 knockout cells were used for eCLIP. (*B*) Microscopic imaging to evaluate the association of endoplasmic reticulum (ER) with purified nuclei. (*Left*) Nuclei after one-time wash with hypotonic buffer containing 0.5% NP40. (*Right*) Nuclei after seven times wash with hypotonic buffer containing 2.0% NP40. (Blue) Nuclear staining with DAPI; (red) endoplasmic reticulum marker. (*C*) Western blot of *AGO1*, *AGO2*, *AGO1/2*, and *AGO1/2/3* knockout cell lines to evaluate the purity of nuclear and cytoplasmic samples and presence of AGO protein variants (representative of *n* = 3). Quality was confirmed for all samples prior to submission for RNA-seq.

The AGO protein variants are functionally redundant in miRNA silencing (Su et al. 2009) suggesting that multiple knockouts are required to achieve clear effects. Therefore, we obtained *AGO1*, *AGO2*, *AGO1/2*, and *AGO1/2/3* knockout cells (Fig. 1A,C; Chu et al. 2020). In HCT116 cells (Liu et al. 2018, 2019) and other cell lines (Petri et al. 2011), AGO4 protein is expressed at barely detectable levels and its knockout was not pursued.

We used purified nuclei for this study. Obtaining sufficiently pure nuclei is necessary because the endoplasmic reticulum contains AGO protein and is contiguous with the nuclear membrane (Stalder et al. 2013). The ER must be removed without disrupting the nuclei. For HCT116 cells, previous protocols (Gagnon et al. 2014a,b) for removing ER from nuclei were not adequate for this cell line and required methodical optimization to identify more effective conditions. Microscopy (Fig. 1B) and western analysis (Fig. 1C) confirmed the removal of proteins associated with the endoplasmic reticulum (ER) and the presence in nuclei of AGO proteins.

We analyzed the nuclear RNAs Neat1 and MALAT1 to evaluate the effectiveness of purifying nuclei and cytoplasmic fractions. Nuclear RNA genes NEAT1 and MALAT1 were enriched ∼11- and ∼18-fold, respectively, in nuclear input samples relative to cytoplasm, while the mRNA-encoding GAPDH was twofold enriched in cytoplasm relative to nuclear samples. We note that our goal was to get as pure nuclei as possible for this experiment. Therefore, relatively harsh lysis conditions unavoidably disrupted a small fraction of cell nuclei, and a small amount of nuclear RNAs such as NEAT1 or MALAT1 may appear in the cytoplasmic fraction. These values, therefore, represent minimum estimates of the relative nuclear localization for these RNAs.

Enhanced crosslinking immunoprecipitation (eCLIP) is a sensitive technique for identifying binding sites between RNA and protein that has been optimized to reduce the potential for artifactual background interactions (Van Nostrand et al. 2016). Successful eCLIP, however, depends on the quality of the antibody used in the immunoprecipitation. An elegant earlier study pointed out that at least one widely used anti-AGO2 antibody was not adequate for reliable immunoprecipitation experiments (Van Eijl et al. 2017).

To minimize the potential for misleading data due to inadequate antibody selectivity, for our primary eCLIP analysis, we chose anti-AGO2 antibody 3148 that had been well characterized as efficient for pull-down experiments (Grey et al. 2010; Boudreau et al. 2014) and that we had previously used for AGO2 eCLIP for cytoplasmic RNA. We identified AGO2 in the purified nuclei of crosslinked wild-type HCT116 cells and confirmed the absence of AGO2 protein in *AGO2* knockout cells. The nuclear samples yielded between 10 and 25 million usable reads, sufficient to provide adequate coverage for thousands of genes (Supplemental Fig. S1).

The use of *AGO2* knockout cells is critical for this analysis—nonselective binding to protein should be observed in both wild-type and AGO2 knockout cells, whereas selective reads should be observed in wild-type cells. After eCLIP and collection of RNA-seq data, we subtract sequence reads detected in the AGO2 knockout cells to further reduce the likelihood that our analysis will be affected by the detection of association between RNA and proteins other than AGO2. The nuclear RNA isolated from AGO2-pull-down in *AGO2* KO cells required five additional PCR cycles to get the same concentration of cDNA as obtained from WT cells. The efficiency of AGO2 pull-down using the 3148 anti-AGO2 antibody (Grey et al. 2010) relative to input and IgG controls was reported in our previous paper examining AGO2 interactions in the cytoplasm (Chu et al. 2020).

### Location of AGO2 within nuclear RNA

eCLIP data yields “clusters” of overlapping sequencing reads that identify potential sites for AGO protein binding within cellular RNA. We had previously reported eCLIP data from the cytoplasm of HCT116 cells, revealing that AGO-binding clusters were primarily localized to the 3′-untranslated region (3′-UTR) (Chu et al. 2020). These data were consistent with standard assumptions about the mechanism of action for miRNAs, AGO protein, and RNAi in which cytoplasmic regulation is achieved by miRNA recognition at sequences within 3′-UTRs.

**FIGURE 2.**
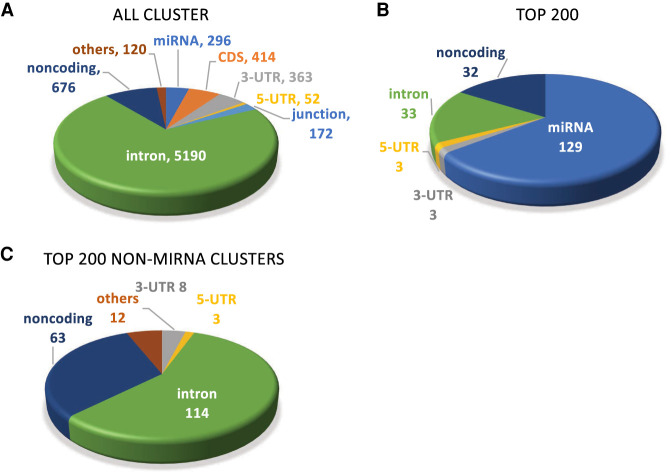
Localization of AGO2 protein engagement with nuclear RNA disclosed by eCLIP using anti-AGO2 antibody 3148. (*A*) Relative distribution of all AGO-binding clusters within nuclear RNA. (*B*,*C*) Relative distribution of top clusters, ranked by (*B*) significance for the top 200 clusters overall and (*C*) the top 200 clusters that that are not directly associated with miRNAs.

For nuclear RNA, however, almost 10-fold more AGO2 protein binding clusters were localized within intronic RNA than within the 3′-UTR (Fig. 2A). Clusters were also detected within noncoding RNA, coding sequence RNA, and 5′-untranslated regions. Clusters were ranked by significance (*P* < 0.05). Of the 200 most significant clusters, 129 were within sequences that encode miRNAs (Fig. 2B). miRNAs are loaded directly onto AGO2, while mRNAs have a less direct association with AGO2. The direct contact between AGO2 and miRNAs, in combination with the relatively high expression of many miRNAs relative to mRNAs, may explain the prevalence of detecting strong miRNA:AGO associations. We had previously observed the dominance of significant miRNA clusters in our analysis of cytoplasmic AGO2-eCLIP samples (Chu et al. 2020). In contrast, in the size-matched input nuclear sample, a majority of the top 200 clusters were noncoding RNAs such as snRNAs and other miscellaneous small RNAs.

To evaluate targets outside of sequences encoding miRNAs, we focused on the top 200 significant clusters that were not miRNAs (Fig. 2C). One hundred and fourteen clusters were within introns and only eight were within 3′-UTRs. The potential consequences of AGO2 binding within introns is discussed below.

### miRNA levels in cell nuclei mirror those in cell cytoplasm

Our laboratory and others have previously reported data from standard RNA-seq that show miRNAs are found in cell nuclei (Jeffries et al. 2011; Gagnon et al. 2014a; Sarshad et al. 2018). To further this analysis, we examined eCLIP-seq data for purified nuclear RNAs and compared that data to our previously published results evaluating eCLIP-seq data derived from cytoplasmic RNA (Chu et al. 2020).

**FIGURE 3.**
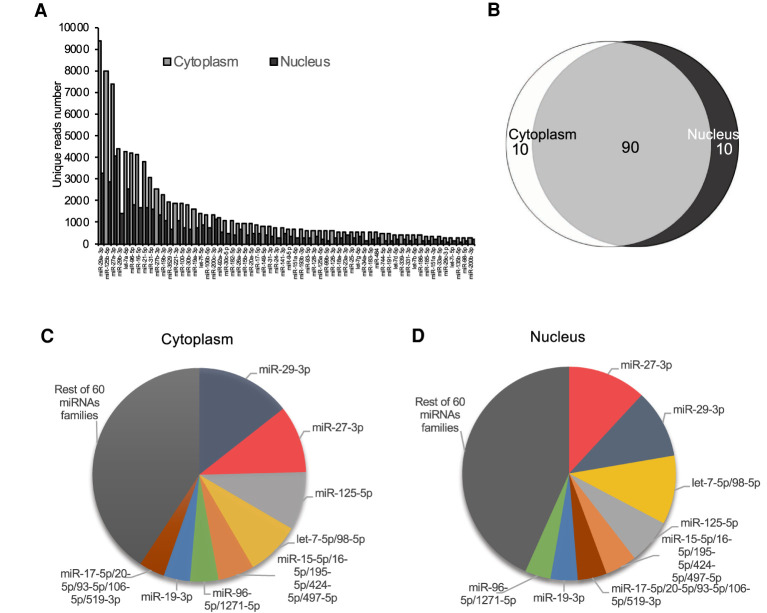
Ranking of AGO2 protein-associated miRNAs in cytoplasm and nucleus according to the number of reads detected during RNA-seq. (*A*) miRNA abundance ranked the top 60 by the read number associated with each miRNA after eCLIP. (*B*) Overlap between the top 100 miRNAs in nuclear and cytoplasmic fractions. (*C*,*D*) Percentage distribution of the top 100 miRNAs, grouped as families in the cytoplasm (*C*) and nucleus (*D*).

We compared the number of eCLIP RNA-seq reads for the 60 most expressed miRNAs in the nucleus to the read number of highly expressed miRNAs in the cytoplasm and observed similar trends in both cell compartments (Fig. 3A). We also observed strong overlap among the 100 miRNAs with the highest read number (Fig. 3B). The top eight miRNA families were the same in the nucleus and the cytoplasm (Fig. 3C,D). These highly ranked miRNAs accounting for ∼60% of all reads were due to miRNAs. It is reasonable to expect that these highly ranked miRNAs will be most likely to have strong biological activities in HCT116 cells.

### Effect of AGO expression on alternative splicing

The predominance of AGO2 protein binding clusters within intronic RNA, in combination with previous observations that synthetic RNAs could target introns to alter splicing (Allo et al. 2009; Liu et al. 2012) led us to examine the hypothesis that AGO2 binding in concert with miRNAs might be an endogenous regulatory mechanism for controlling alternative splicing. We integrated our AGO2-eCLIP-seq and RNA-seq data to identify genes that would be optimal candidates for detailed investigation of endogenous RNAi in the nucleus.

**FIGURE 4.**
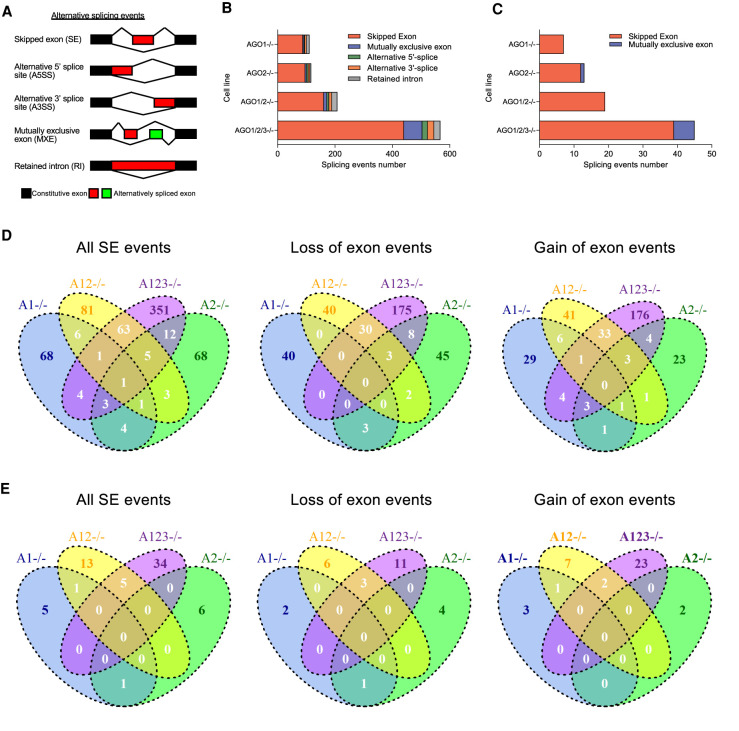
The effect of *AGO* knockouts on alternative splicing events. (*A*) Scheme showing various types of alternative splicing events. (*B*) Alternative splicing events observed upon *AGO1*, *AGO2*, *AGO1/2*, *AGO1/2/3* knockout. (*C*) Alternative splicing events upon *AGO1*, *AGO2*, *AGO1/2*, *AGO1/2/3* knockout associated with nearby AGO2 protein-binding sites detected by eCLIP within introns. (*D*) VENN diagrams showing the overlap in skipped exon events for the *AGO1*, *AGO2*, *AGO1/2*, and *AGO1/2/3* knockout cell lines. (*E*) VENN diagrams showing the overlap of alternative splicing events in *AGO1*, *AGO2*, *AGO1/2*, and *AGO1/2/3* knockout cell lines for genes that have intronic binding sites for AGO2 detect by eCLIP.

The impact on the total number of alternative splicing events was measured, including skipped exons, mutually exclusive exons, alternative 5′ splice sites, and alternative 3′ splice sites (Fig. 4A). In the initial analysis, changes in the *AGO* knockout cell lines relative to wild-type cells were evaluated. We subsequently evaluated changes in splicing at genes with significant AGO2 binding clusters within introns relative to AGO knockout cells serving as negative controls or cells serving as size-matched input controls. We observed hundreds of changes in splicing (Fig. 4B), but less than 50 changes were associated with AGO2 binding clusters within introns (Fig. 4C).

Experimental validation of results from large data sets is time consuming and it is important to prioritize candidate genes for analysis. Some splicing events are likely to be indirect effects or noise unrelated to knocking out the *AGO* variants. We reasoned that the splicing events that are most likely to be endogenously regulated by RNAi would show significant AGO2 occupancy and be shared by more than one *AGO* knockout cell line, especially between the *AGO1/2* double knockout and the *AGO1/2/3* triple knockout.

We observed that for loss or gain of exon events, several dozen significant events were shared between the *AGO1/2* and *AGO1/2/3* knockout cells (Fig. 4D). For splicing events associated with location for AGO2 protein binding to introns and shared by *AGO1/2* and *AGO1/2/3* cells, we observed five skipped exon events, three losses of exon events, and two gains of exon events (Fig. 4E). These splicing events identified through the combination of eCLIP and RNA-seq are the strongest candidates for endogenous miRNA-mediated nuclear gene regulation.

Based on the precedent supplied by splice-modulating antisense oligonucleotides (Havens and Hastings 2016), the simplest explanation is that miRNAs, by binding near splice sites and recruiting AGO and related proteins, block binding sites for splicing factors. We examined the 39 alternative splicing events that were associated with AGO2 binding in wild-type relative to AGO1/2/3 knockout cells (Fig. 4C,E). To evaluate the hypothesis that splicing changes were associated with changes to the association of RNA binding proteins, we evaluated protein binding events noted in dbCLIP, the database of information from CLIP studies of RNA binding proteins including splicing factors (Yang et al. 2015; Zhu et al. 2019).

Association of RNA binding proteins was enriched near AGO2 binding sites (Supplemental Fig. 2A). No binding sites were reported to be directly adjacent to the AGO2 sites within the RUBCN and FKBP14 intronic RNAs, but there were several binding sites within 200 nt (Supplemental Fig. 2B). Of the 39 candidate sites, 13 sites directly overlapped or bound <10 nt apart from AGO2-binding sites (Supplemental Fig. 2C). While not conclusive because of the limited number of splicing factors that have been analyzed using CLIP methodology, this information from dbCLIP offers some support for a plausible “blocking” mechanism of action.

### Experimental validation of splicing changes induced by AGO knockouts

**FIGURE 5.**
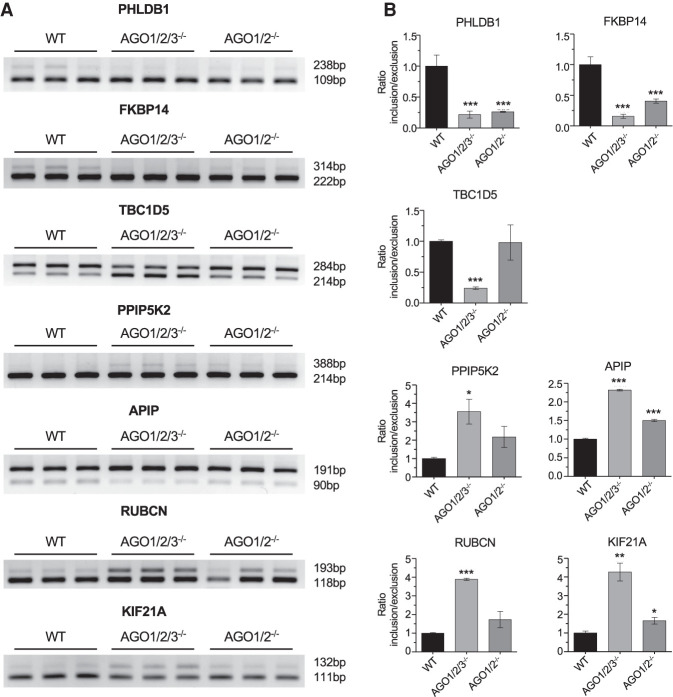
Validating the effect of *AGO* knockouts on alternative splicing. (*A*) Semiquantitative PCR validation of skipped exon events in *AGO1/2/3* KO and *AGO1/2 KO* cells. (*B*) Quantitation of data shown in part *A*. Error bars represent standard deviation (SD). (*) *P* < 0.05; (**) *P* < 0.01; (***) *P* < 0.001 compared with WT by one-way ANOVA and Dunnett's multiple comparisons test.

We chose several splicing events for experimental validation based on their overlap in both *AGO1/2* and *AGO1/2/3* knockout cells (Fig. 4E; Supplemental Fig. S3A–G). These included exon exclusion events for *PHLDB1*, *FKBP14*, and *TBC1D5*, and exon inclusion events for *PPIP5K2*, *APIP*, *RUBCN*, and *KIF21A*. In all cases, we confirmed the alternative splicing predicted by our RNA-seq data (Fig. 5). Splicing changes were confirmed using a second PCR primer set for amplification (Supplemental Fig. S4) for all targets except *KIF21A*, in which case we were not able to identify a second priming set because of the small (21 base-pair difference in length between the two isoforms).

For three genes, *PHLDB1*, *FKBP14*, and *APIP,* the experimentally determined difference in splicing was observed in both the *AGO1/2* and *AGO1/2/3* knockout cells. For the other four genes, the difference in splicing was observed only in the triple knockout cells, but not the *AGO1/2* double knockout cells, possibly reflecting the impact of the remaining AGO3 protein.

In an accompanying study, we examined the effect of knocking out *TNRC6*, a gene that produces three paralog scaffolding proteins (TNRC6A, TNRC6B, and TNRC6C) that interact in concert with AGO2 protein. RNA-seq from *TNRC6* knockout cells revealed many of the same splicing changes, and qPCR validation yielded splicing changes in *PHLDB1*, *FKBP14*, *RUBCN*, *KIF21A*, and *TBC1D5* (Johnson et al. 2021).

### Effect of *DROSHA* knockout on splicing of select genes

**FIGURE 6.**
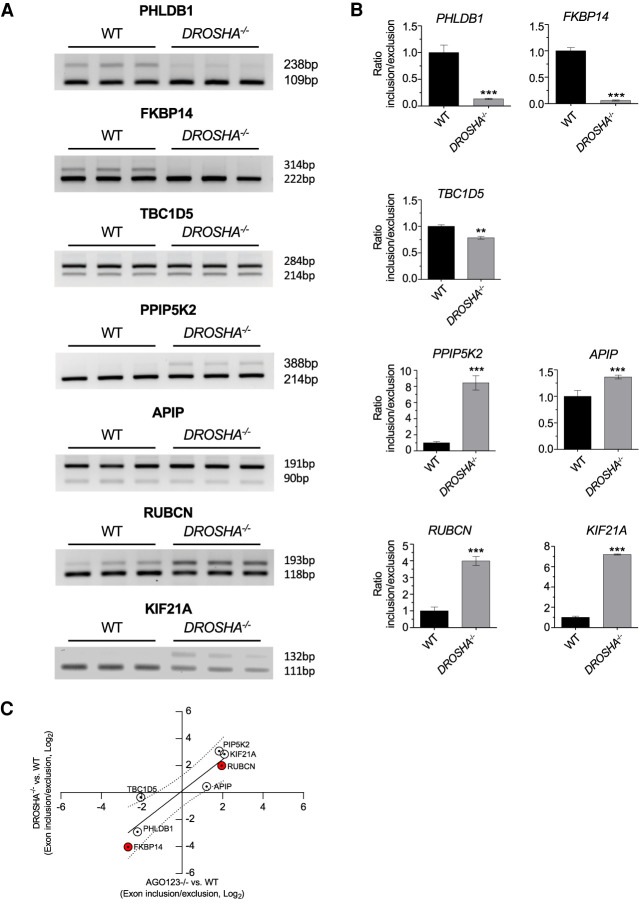
Effect of *DROSHA* knockout on genes identified as candidates from AGO2 eCLIP and RNA-seq of *AGO* knockout cells. Skipped exon events in *DROSHA* KO cells. (*A*) Semiquantitative PCR validation of skipped exon events in *DROSHA* KO cells. (*B*) Quantitation of data shown in part *A*. Error bars represent standard deviation (SD). (*) *P* < 0.05; (**) *P* < 0.01 compared with WT by *t*-test. (*C*) Scatter plot showing the exon inclusion/exclusion ratio between *AGO1/2/3 KO* and *DROSHA KO*. Each point represents average of three biological replicates. (Dashed line) 95% confidence interval. (Solid line) Best fit. *RUBCN* and *FKBP14* were subject to further experimental validation (Fig. 7) and are highlighted in red.

DROSHA is an RNase III enzyme that catalyzes consecutive processing events during miRNA biogenesis (Michlewski and Caceres 2019; Treiber et al. 2019). Like the AGO protein variants, DROSHA is an integral component of regulation by miRNAs. Knockout of DROSHA protein would be expected to have similar effects on gene expression and would tend to confirm the impact on splicing that we observe is due to impairing regulation by RNAi. We obtained *DROSHA* knockout cells (Supplemental Fig. S5) and examined the impact of loss of *DROSHA* (Fig. 6) on splicing of the seven genes chosen for experimental validation in our *AGO* knockout cells (Fig. 5).

For five of the seven genes, we observed changes in alternative splicing consistent with our *AGO* knockout RNA-seq data (Fig. 4) and our experimental PCR measurement of splicing changes after *AGO* knockout (Fig. 5; Supplemental Fig. S4). We observed exon exclusion events for *PHLDB1* and *FKBP14* and exon inclusion events for *PPIP5K2*, *RUBCN*, and *KIF21A* (Fig. 6). The alternative splicing changes for these five genes were confirmed using a second primer set for amplification (Supplemental Fig. S6). The splicing changes showed a clear correlation between *AGO1/2/3* and *DORSHA* knockout cells (*r* = 0.86; *P* = 0.01) (Fig. 6C; Supplemental Fig. S7), consistent with knockdown of genes in a common pathway. Our similar data from loss of proteins from three different families (AGO, DROSHA, TNRC6) in the RNAi pathway support the hypothesis that the RNAi machinery can regulate alternative splicing.

### Alteration of splicing by synthetic miRNA mimics and anti-miRs

**FIGURE 7.**
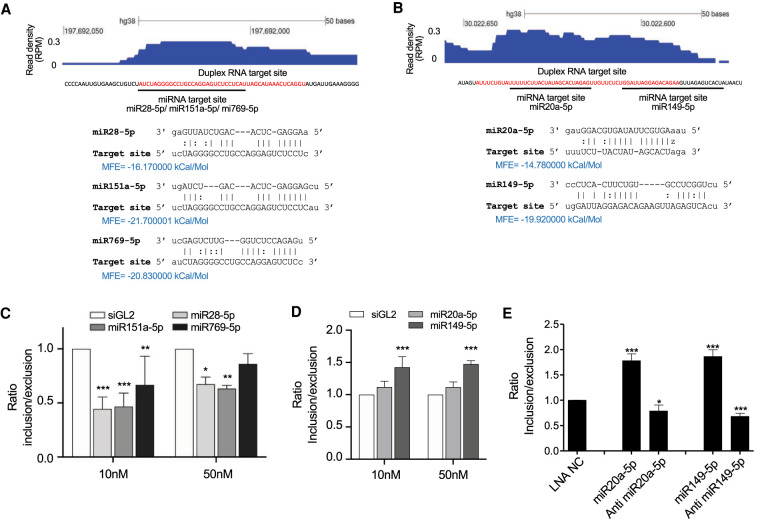
miRNA mimics induce splicing changes in *RUBCN* and *FKBP14*. (*A*) AGO2 protein binding cluster within *RUBCN* intronic RNA and potential binding sites for miRNAs. (*B*) AGO2 cluster within *FKBP14* intronic RNA and potential binding sites for miRNAs. Red colored sequence defines the AGO2 clusters identified from eCLIP-seq. (*C*,*D*) qPCR quantification of splicing changes after one cay by miRNA mimics in (*C*) *RUBCN* and (*D*) F*KBP14*. (*E*) qPCR quantification of splicing changes after four cays by anti-miR LNAs or miRNA mimics within *FKBP14*. Error bars represent standard deviation (SD). (*) *P* < 0.05; (**) *P* < 0.01; (***) *P* < 0.001 compared with WT by one-way ANOVA and Dunnett's multiple comparisons test. siGL2 is negative control siRNA. LNA-NC is a negative control ASO. (MFE) Minimum free energy. Experiments with miRNA mimics required a 1 d incubation with the synthetic nucleic acid; experiments with anti-miRs required 4 d.

We used synthetic miRNA mimics and anti-miRs (Supplemental Table S1) to test the link between RNA recognition within introns and regulation of alternative splicing. We examined splicing clusters at the *RUBCN* and *FKBP14* genes for complementarity to miRNAs (Fig. 7A,B). To predict miRNAs that might bind at those AGO2 clusters, two criteria were set: (i) miRNAs were limited to the top 100 most highly ranked miRNAs from our RNA-seq analysis; and (ii) the minimum free energy (MFE) for matching at the site was required ≤14 Kcal/mol.

From this list of miRNA candidates, we focused on miRNAs that overlapped intronic AGO2-binding clusters that showed significant splicing changes. We identified three miRNAs for *RUBCN* (miR28-5p, miR151a-5p, miR769-5p) and two for *FKBP14* (miR20a-5p, miR149-5p). We observed that miRNA mimics decreased exon inclusion when targeting *RUBCN* and increased exon inclusion when targeting *FKBP14* (Fig. 7C,D).

Anti-miRs are synthetic oligonucleotides that are complementary to miRNAs and have the potential to block their effects (Krutzfeldt et al. 2005). We introduced locked nucleic acid (LNA) anti-miRs into HCT116 cells that were designed to target two miRNAs, miR-20 and miR-149 with complementarity to the AGO2-protein binding cluster within *FKBP14* intronic RNA. Both anti-miRs reduce exon inclusion, while addition of miRNA mimics in parallel yield the opposite effect (Fig. 7E). Taken together, these modest effects are not conclusive but do support the potential for miRNAs to influence splicing.

## DISCUSSION

### Localizing AGO2 binding within the nuclear transcriptome

We had previously used CLIP-seq (Chi et al. 2009; Hafner et al. 2010) to identify AGO protein binding sites but could not unambiguously validate any expression changes (results not shown). We found that roadblocks to successful application of CLIP-seq in these prior experiments included: (i) We were comparing wild-type cells to cells where AGO2 expression had been reduced by siRNA-mediated knockdown. Since the knockdown was not complete, residual AGO2 protein might confound our ability to interpret data. (ii) The expression of the other AGO variants was not reduced, retaining a pool of AGO available for controlling gene expression. (iii) While CLIP-seq is a powerful approach, artifactual identification of AGO binding clusters can occur. Experimentally validating clusters is time consuming, and we found that discriminating among clusters to identify the most promising candidates for analysis was inefficient.

For a novel cellular process like small RNA mediated control of splicing, a more defined experimental strategy was necessary. To better discriminate against background in both CLIP and RNA-seq experiments, we obtained *AGO2* knockout cells (Golden et al. 2017; Chu et al. 2020). We also obtained *AGO1/2* and *AGO1/2/3* knockout cells (Chu et al. 2020) to remove the three most highly expressed AGO protein variants. Finally, we used an improved CLIP-seq technique, eCLIP, to reduce background signal (Van Nostrand et al. 2016).

### Nuclear localization of miRNAs

Our eCLIP data revealed that the strongest and most significant AGO2 protein binding clusters within nuclear RNA were associated with miRNAs (Fig. 2). Strong association with miRNAs is probably due to the AGO:miRNA interaction being direct instead of the secondary interaction formed between AGO and mRNA.

Our data demonstrate that AGO2-bound miRNAs in HCT116 cell nuclei have abundances like those in cell cytoplasm. There is no evidence in HCT116 cells of dramatic differences in miRNA distribution, suggesting that there is no need to hypothesize mechanisms to preferentially shuttle select individual miRNAs to nuclei relative to cytoplasm or vice versa in this context. The presence of RNAi factors (Robb et al. 2005; Gagnon et al. 2014a) and miRNAs (at a similar abundance) in both cytoplasm, and nuclei supports the conclusion that the RNAi has the potential to recognize nuclear RNA and control gene expression and that there may be similar rules governing the recognition of target sequences.

Our data from one cell line grown under standard conditions does not rule out the possibility that a miRNA might be preferentially localized to nucleus relative to cytoplasm or vice versa. However, the data do suggest that compelling evidence should be shown to support the shuttling of individual miRNAs.

### Impact of AGO expression on alternative splicing

After miRNAs, the most prevalent AGO2 protein association was within intronic RNA (Fig. 2). This association between AGO2 and introns is consistent with previous observations (Moore et al. 2015; Sarshad et al. 2018). We and others had previously shown that synthetic duplex RNAs complementary to introns could be used to control splicing (Allo et al. 2009; Liu et al. 2012), leading to the hypothesis that endogenous miRNAs might also be involved in the control of alternative splicing. The obvious mechanism for controlling splicing would be for the AGO:miRNA complex to block recognition of splicing factors. This mechanism is widely used for antisense oligonucleotides that modulate splicing (Havens and Hastings 2016), and we had previously observed that duplex RNAs mimic the sequence of splice-modulating antisense oligonucleotides and affect alternative splicing (Liu et al. 2012).

We now report several observations that support the hypothesis that small RNAs can control splicing. When *AGO* expression is knocked out, we observe several hundred changes in alternative splicing. Dozens of these changes occur at genes that have significant clusters of sequencing reads overlapping nearby introns. We further prioritized candidates by requiring that they be observed in both *AGO1/2* and *AGO1/2/3* knockout cells.

The significant splicing changes that were detected by RNA-seq were experimentally validated by RT-PCR. Those same genes showed similar changes in splicing upon knockout of DROSHA, a processing enzyme that is an upstream component of the RNAi machinery. TNRC6 is a critical RNAi scaffolding protein that binds to AGO, and several of these top candidate genes also showed splicing changes in TNRC6 knockout cells (see Johnson et al. 2021). Finally, miRNA mimics and miRNA inhibitors (anti-miRs) that target potential miRNA binding sites within introns were shown to regulate alternative splicing. Taken together, these data support the conclusion that miRNAs and the endogenous cellular RNAi machinery can regulate alternative splicing in HCT116 cells.

Some miRNAs are encoded by introns, and we used dbCLIP data to examine the hypothesis that binding of AGO2 and associated miRNA processing enzymes to intron-encoding RNA sequences might be responsible for some of the splicing changes that we observed. Based on the mirtronDB database (Da Fonseca et al. 2019) and the snoRNABase and miRBase tracks in the genome browser, none of the alternative splicing events associated with AGO2 binding sites overlapped the encoding regions of miRNAs. In addition, DGCR8 (a partner protein of DROSHA) did not appear to bind near the 39 AGO2 binding events associated with changes in alternative splicing in wild-type versus AGO1/2/3.

While our data suggest that miRNA mimics can regulate alternative splicing, the number of genes affected was modest. Changes in only 70 alternative splicing events are observed in both *AGO1/2* and *AGO1/2/3* knockout cells. For alternative splicing events that are associated with significant AGO binding clusters and observed in both *AGO1/2* and *AGO1/2/3* knockout cells, we observe just five skipped exon events, three loss of exon events, and two gain of exon events. A similar small number of events are observed in *TNRC6* knockout cells (see Johnson et al. 2021).

Why were so few events detected? We do not claim that we have identified all alternative splicing events that are candidates for regulation by miRNAs. For example, intronic RNA that is present at low copy numbers might be biologically significant but remain undetected during eCLIP. Our criteria for selecting candidates (significantly altered splicing in both *AGO1/2/3* and *AGO1/2/3* knockout cells for genes with significant intronic AGO2 protein binding clusters) was stringent and might also have led us to miss biologically relevant alternative splicing events.

Our HCT116 cells were grown under standard, permissive cell culture conditions. It is also possible that under standard cell culture conditions, the impact of the cellular RNA machinery and the number of RNA-regulated splicing events is small. miRNAs may exert their most robust effects under a limited range of environmental or growth conditions. miRNAs that control splicing would be no exception to this model—a potentially powerful regulatory mechanism that becomes important at critical points during development, response to environment change, or disease pathogenesis. If true, the modest changes in splicing that we observe in HCT116 cells might hint at the possibility that a larger range of changes occurs in other settings.

### Conclusions

AGO:miRNA complexes are ribonucleoproteins that have the potential to recognize sequences throughout the transcriptome. This ability coupled with the presence of both AGO proteins and miRNAs in the nucleus suggests the potential to control gene splicing. Consistent with this hypothesis, we observe AGO2 binding within intronic RNA and identify endogenous miRNAs that may affect alternative splicing. Our data expand the potential for RNAi to control gene expression and miRNAs that affect splicing may play significant roles in physiology and disease.

## MATERIALS AND METHODS

### Cell lines

The HCT116 cell line (Horizon Discovery) originated from the American Type Culture Collection (ATCC). ATCC authenticated this HCT116 cell line using Short Tandem Repeat (STR) analysis as described (Capes-Davis et al. 2012). The ATCC STR analysis compared seventeen short tandem repeat loci plus the gender determining locus, Amelogenin, to verify the HCT116 cell line (ATCC CCL 247). The European Collection of Authenticated Cell Cultures (ECACC) performed an additional STR analysis of seventeen loci on the cells received from ATCC, and the verified HCT116 cells (ECACC 91091005) were supplied to Horizon Discovery for distribution.

For eCLIP, we used HCT116:AGO2 knockout cells obtained from Joshua T. Mendell (UT Southwestern). The *AGO1*, *AGO2*, *AGO1/2*, and *AGO1/2/3* knockout cell lines used for RNA-seq were prepared using GenCRISPR gene editing technology and services (GenScript) and verified HCT116 cells (Horizon Discovery). The AGO2 knockout cell line was independently generated to ensure that all cell lines used for RNA-seq were derived from similar genetic backgrounds. gRNA sequence, gRNA target locations, and DNA sequencing results have been reported previously (Chu et al. 2020). The *DROSHA* knockout cell lines used were prepared using GenCRISPR gene editing technology and services (GenScript) and verified HCT116 cells (Horizon Discovery). All HCT116 and HCT116-derived cells were cultured in McCoy's 5A Medium (Sigma-Aldrich) supplemented with final 10% FBS at 37°C in 5% CO_2_. For the cell growth assay, the cells were seeded at a density of 50,000 cells/mL, disassociated with 1× trypsin, and counted using trypan blue staining (TC20 Automated Cell Counter, Bio-Rad).

### Preparation of nuclear extract

Nuclear extract isolation was similar to that previously described (Gagnon et al. 2014a,b), although modifications to adapt the protocol for HCT116 and HCT116-derived cells were required. Cells at ∼95% confluence were lysed in hypotonic lysis buffer (HLB) (10 mM Tris-HCl, pH-7.4, 10 mM NaCl, 3 mM MgCl_2_, 2.5% NP-40, 0.5 mM DTT, 1× protease inhibitor [Roche], 50 U/mL ribonuclease inhibitors [RNasin Plus, Promega]), and supernatant collected as cytoplasmic fraction. Western blots to determine the purity of fractions and RNAi factors distribution were performed using western blot analysis as before (Gagnon et al. 2014a,b).

### Western blot analysis

Total protein lysate was prepared resuspending cells in lysis buffer (50 mM Tris-HCl, pH-7.0, 120 mM NaCl, 0.5% NP-40, 1 mM EDTA, 1 mM DTT, 1× protease inhibitor [Roche, cOmplete]). Proteins were separated on 4%–20% gradient Mini-PROTEAN TGX Precast Gels (Bio-Rad). After gel electrophoresis, proteins were wet transferred to nitrocellulose membrane (0.45 µm, GE Healthcare Life Sciences) at 100 V for 75 min. Membranes were blocked for 1 h at room temperature with 5% milk in 1× PBS containing 0.05% TWEEN-20. Blocked membranes were incubated with the primary antibodies in blocking buffer at 4°C on rocking platform overnight: using anti-AGO1, 1:2000 (5053, Cell Signaling), anti-AGO2, 1:1500 (015-22031, Fujifilm WAKO), anti-AGO3, 1:500 (39787, Active Motif), anti-Calnexin, 1:1000 (2433, Cell Signaling), anti-LaminA/C, 1:1500 (ab8984, Abcam), anti-β-Tubulin, 1:5000 (T5201, Sigma-Aldrich), anti-Histone H3, 1:20,000 (2650S, Cell Signaling) antibodies. After primary antibody incubation, membranes were washed 3 × 10 min at room temperature with 1× PBS + 0.05% TWEEN-20 (PBST 0.05%) and then incubated for 1 h at room temperature with respective secondary antibodies in blocking buffer. Membranes were washed again 4 × 10 min in PBST 0.05%. Washed membranes were soaked with HRP substrate according to manufacturer's recommendations (SuperSignal West Pico Chemiluminescent substrate, Thermo Scientific) and exposed to films. The films were scanned, and bands were quantified using ImageJ software.

### Cell transfection

For experiments of miRNA mimics and anti-miR LNAs, cells were plated onto 48-well plates at 30,000 cells per well 1 d prior to transfection. Cells were transfected with double-strand RNAs or miRNA mimics by using Lipofectamine 3000 (Invitrogen). Detailed sequence information is shown in Supplemental Table S4. miRCURY LNATM miRNA inhibitor for anti-miR20a-5p and anti-miR149-5p were bought from Qiagen. Total RNAs were extracted 24 h or 96 h (for anti-miR test) after transfection with TRIzol (Invitrogen) for RT-PCR.

### Splicing analysis by gel electrophoresis and qPCR

Total RNA was extracted from HCT116 wild-type, knockout cells, and transfected cells, and treated with DNase I (Worthington Biochemical) at 25°C for 20 min, 75°C for 10 min. Reverse transcription was performed using the High-Capacity Reverse Transcription kit (Applied Biosystems) per the manufacturer's protocol. Two micrograms of total RNA was used per 20 µL of reaction mixture. In gel electrophoresis analysis, PCR amplification was as follows: 95°C 5 min and 95°C 15 sec, 60°C 1 min for 38 cycles. The PCR products were separated by 1.5% agarose gel electrophoresis. The bands were quantified by using ImageJ software. In qPCR analysis for splicing changes by using double-strand RNAs and miRNA mimics, PCR was performed on a 7500 real-time PCR system (Applied Biosystems) using iTaq SYBR Green Supermix (Bio-Rad). PCR reactions were done in triplicates at 55°C 2 min, 95°C 3 min, and 95°C 20 sec, 60°C 1 min for 40 cycles in an optical 96-well plate. The expression level was compared between exon inclusion variants and exon exclusion variants. PCR primers are shown in Supplemental Table S2.

### RNA-seq for gene expression analysis

WT HCT116, AGO1, AGO2, AGO1/2, and AGO1/2/3 knockout cells were used for RNA-seq. Three biological replicated samples were sequenced. Approximately 3.0 × 10^6^ cells were seeded in a 15-cm large dish. Cells were harvested 48 h later, and RNA was extracted from cytoplasmic or nuclear fractions using the RNeasy Mini Kit (Qiagen) with an on-column DNase digestion. Sequencing libraries were generated using the TruSeq Stranded Total RNA with Ribo-Zero Human/ Mouse/Rat Low-throughput (LT) kit (Illumina) and run on a NextSeq 500 for paired-end sequencing using the NextSeq 500/550 High Output v2 Kit, 150 cycles (Illumina).

Quality assessment of the RNA-seq data was done using NGS-QC-Toolkit43 with default settings. Quality-filtered reads generated by the tool were then aligned to the human reference genome hg38 and transcriptome gencode v75 using the STAR (v 2.5.2b) with default settings. Read counts obtained from STAR were used as input for Salmon (v 1.0.0) and Deseq2 for gene differential expression analysis. Genes with adjusted *P* ≤ 0.05 were regarded as differentially expressed for comparisons of each sample group.

### eCLIP

Control and AGO2^−/−^ HCT116 cells (obtained from Dr. Joshua Mendel, UT Southwestern) were seeded in 15 cm dishes with 12 dishes per cell line at 3.0 × 10^6^ cells per dish. Cells were cultured for 48 h and subsequently UV crosslinked at 300 mJ/cm^2^. Nuclear fraction was collected as described above. eCLIP was performed using the frozen samples as previously described (Van Nostrand et al. 2016), using anti-AGO2 antibody for IPs (3148, gift from the Jay A. Nelson laboratory). For each cell line, duplicate input and IP samples were prepared and sequenced. The RiL19 RNA adapter (Supplemental Table S3) was used as the 3′ RNA linker for input samples. RNA adapters RNA_A01, RNA_B06, RNA_C01, RNA_D08, RNA_X1A, RNA_X1B, RNA_X2A, RNA_X2B were used for IP samples (Supplemental Table S3). PAGE purified DNA oligonucleotides were obtained from IDT for the PCR library amplification step (Supplemental Table S3). PCR amplification was performed using between 11 and 16 cycles for all samples. Paired-end sequencing was performed on a NextSeq 500 using the NextSeq 500/550 High Output v2 Kit, 100 cycle (Illumina). eCLIP data was analyzed as previously described (Chu et al. 2020). Initial AGO2 binding clusters identified by CLIPper (Lovci et al. 2013) in wild-type HCT116 were filtered to keep only clusters that are statistically significant (*P* < 0.001). For each region, normalization to total usable reads was performed and a fold change between IP and combined samples (input and IP in knockout cell line samples) was calculated. Significant CLIP clusters in each data set were defined by (i) *P* < 0.05 determined by the Fisher exact test or Yates’ χ^2^ test, and (ii) log_2_ fold change of normalized reads in the cluster was ≥2 comparing IP to combined (input + IP in knockout cells).

The final CLIP clusters for AGO2 were identified by first identifying significant clusters present in both experimental replicates. A cluster was considered to be present in both replicates if it occurred on the same strand and the replicate clusters overlapped by at least 1/3 of their total length. Significant clusters from both replicates were then merged to define the final cluster length. Clusters were annotated based on their genomic locations (Gencode v27). If a cluster was assigned to multiple annotations, the annotation was selected using the following priority: CDS exon > 3′-UTR > 5′ UTR > Protein-coding gene intron > Noncoding RNA exon > Noncoding RNA intron > Intergenic.

### Estimation of miRNAs expression levels from eCLIP-seq

Bowtie2 was used to map the miRNA tags to the miRBase (mature miRNAs), allowing up to one mismatch. The miRNA expression levels are quantified as the number of reads mapped to individual miRNAs normalized by the total number of mapped reads in miRBase. The expression levels from different samples were further normalized by quantile normalization to control for batch effect.

### Statistical analysis

The dynamic and bar graphs represent mean and standard deviation. The averages among cells were compared using one- or two-way analysis of variance followed by Bonferroni and Tukey post-hoc tests (*P* < 0.05). To determine correlation between AGO2 binding cluster significance level and gene expression change in AGO knockout cells, we first tested data sets for normal distribution (D'Agostino and Pearson omnibus normality test, Kolmogorov–Sminov test). Both correlating data sets could not pass the normality test, or in many cases the relation was not linear, therefore we calculated Spearman's correlation coefficient.

## DATA DEPOSITION

All high-throughput sequencing data generated for this study (RNA-seq, eCLIP) have been deposited in the Gene Expression Omnibus under accession number GSE161559.

## SUPPLEMENTAL MATERIAL

Supplemental material is available for this article.

## Supplementary Material

Supplemental Material

## References

[RNA078707CHUC1] Allo M, Buggiano V, Fededa JP, Petrillo E, Schor I, de la Mata M, Agirre E, Plass M, Eyras E, Elela SA, 2009. Control of alternative splicing through siRNA-mediated transcriptional gene silencing. Nat Struct Mol Biol 16: 717–724. 10.1038/nsmb.1620 19543290

[RNA078707CHUC2] Ameyar-Zazoua M, Rachez C, Souidi M, Robin P, Fritsch L, Young R, Morozova N, Fenouil R, Descostes N, Andrau JC, 2012. Argonaute proteins couple chromatin silencing to alternative splicing. Nat Struct Mol Biol 19: 998–1004. 10.1038/nsmb.2373 22961379

[RNA078707CHUC3] Bartel DP. 2018. Metazoan microRNAs. Cell 173: 20–51. 10.1016/j.cell.2018.03.006 29570994 PMC6091663

[RNA078707CHUC4] Boudreau RL, Jiang P, Gilmore BL, Spengler RM, Tirabassi R, Nelson JA, Ross CA, Xing Y, Davidson BL. 2014. Transcriptome-wide discovery of microRNA binding sites in human brain. Neuron 81: 294–305. 10.1016/j.neuron.2013.10.062 24389009 PMC4108341

[RNA078707CHUC5] Capes-Davis A, Reid YA, Kline MC, Storts DR, Strauss E, Dirks WG, Drexler HG, MacLeod RA, Sykes G, Kohara A, 2012. Match criteria for human cell line authentication: Where do we draw the line? Int J Cancer 132: 2510–2519. 10.1002/ijc.27931 23136038

[RNA078707CHUC6] Chi SW, Zang JB, Mele A, Darnell RB. 2009. Argonaute HITS-CLIP decodes microRNA-mRNA interaction maps. Nature 460: 479–486. 10.1038/nature08170 19536157 PMC2733940

[RNA078707CHUC7] Chu Y, Kilikevicius A, Liu J, Johnson K, Yokota S, Corey DR. 2020. Argonaute binding within 3′-untranslated regions poorly predicts gene repression. Nucleic Acids Res 48: 7439–7453. 10.1093/nar/gkaa478 32501500 PMC7367155

[RNA078707CHUC8] Da Fonseca BHR, Domingues DS, Paschoal AR. 2019. mirtronDB: a mitron knowledge base. Bioinformatics 36: 3873–3874. 10.1093/bioinformatics/btz153 PMC6761972 30874795

[RNA078707CHUC9] Gagnon KT, Li L, Chu Y, Janowski BA, Corey DR. 2014a. RNAi factors are present and active in human cell nuclei. Cell Rep 6: 211–221. 10.1016/j.celrep.2013.12.013 24388755 PMC3916906

[RNA078707CHUC10] Gagnon KT, Li L, Janowski BA, Corey DR. 2014b. Analysis of nuclear RNA interference in human cells by subcellular fractionation and argonaute loading. Nat Protoc 9: 2045–2060. 10.1038/nprot.2014.135 25079428 PMC4251768

[RNA078707CHUC11] Ghandi M, Huang FW, Jane-Valbuena J, Kryukov GV, Lo CC, McDonald ER, Barretina J, Gelfand ET, Bielski CM, Li H, 2019. Next-generation characterization of the cancer cell line encyclopedia. Nature 569: 503–508. 10.1038/s41586-019-1186-3 31068700 PMC6697103

[RNA078707CHUC12] Golden RJ, Chen B, Li T, Braun J, Manjunath H, Chen X, Wu J, Schmid V, Chang TC, Kopp F, 2017. An Argonaute phosphorylation cycle promotes microRNA-mediated silencing. Nature 542: 197–202. 10.1038/nature21025 28114302 PMC5302127

[RNA078707CHUC13] Grey F, Tirabassi R, Meyers H, Wu G, McWeeney S, Hook L, Nelson JA. 2010. A viral microRNA down-regulates multiple cell cycle genes through mRNA 5′UTRs. PLoS Pathog 6: e1000967. 10.1371/journal.ppat.1000967 20585629 PMC2891821

[RNA078707CHUC14] Hafner M, Landthaler M, Burger L, Khorshid M, Hausser J, Berninger P, Rothballer R, Ascano M, Jungkamp A-C, Munschauer M, 2010. Transcriptome-wide identification of RNA-binding protein and microRNA target sites by PAR-CLIP. Cell 141: 129–141. 10.1016/j.cell.2010.03.009 20371350 PMC2861495

[RNA078707CHUC15] Havens MA, Hastings M. 2016. Splice-switching antisense oligonucleotides as therapeutic drugs. Nucleic Acids Res 44: 6549–6563. 10.1093/nar/gkw533 27288447 PMC5001604

[RNA078707CHUC16] Huang V, Qin Y, Wang J, Wang X, Place RF, Lin G, Lue TF, Li L-C. 2010. RNAa is conserved in mammalian cells. PLoS One 5: e8848. 10.1371/journal.pone.0008848 20107511 PMC2809750

[RNA078707CHUC17] Janowski BA, Huffman KE, Schwartz JC, Ram R, Hardy D, Shames DS, Minna JD, Corey DR. 2005. Inhibiting gene expression at transcription start sites in chromosomal DNA with antigene RNAs. Nat Chem Biol 1: 216–222. 10.1038/nchembio725 16408038

[RNA078707CHUC18] Janowski BA, Younger ST, Hardy DB, Ram R, Huffman KE, Corey DR. 2007. Activating gene expression in mammalian cells with promoter-targeted duplex RNAs. Nat Chem Biol 3: 166–173. 10.1038/nchembio860 17259978

[RNA078707CHUC19] Jeffries CD, Fried HM, Perkins DO. 2011. Nuclear and cytoplasmic localization of neural stem cell miRNAs. RNA 17: 675–686. 10.1261/rna.2006511 21363885 PMC3062178

[RNA078707CHUC20] Johnson ST, Chu Y, Liu J, Corey DR. 2021. Impact of scaffolding protein TNRC6 paralogs on gene expression and splicing. RNA 27: 1004–1016 (this issue). 10.1261/rna.078709.121 PMC8370741 34108231

[RNA078707CHUC21] Kalantari R, Chiang CM, Corey DR. 2016. Regulation of mammalian transcription and splicing by nuclear RNAi. Nucleic Acids Res 44: 524–537. 10.1093/nar/gkv1305 26612865 PMC4737150

[RNA078707CHUC22] Kim DH, Saetrom P, Snøve O, Rossi JJ. 2008. MicroRNA-directed transcriptional gene silencing in mammalian cells. Proc Natl Acad Sci 105: 16230–16235. 10.1073/pnas.0808830105 18852463 PMC2571020

[RNA078707CHUC23] Krutzfeldt J, Rajewsky N, Braich R, Rajeev KG, Tushl T, Manoharan M, Soffel M. 2005. Silencing of microRNAs in vivo with antagomirs. Nature 438: 685–689. 10.1038/nature04303 16258535

[RNA078707CHUC24] Li LC, Okino ST, Zhao H, Pookot D, Place RF, Urakami S, Enokida H, Dahiya R. 2006. Small RNAs induce transcriptional activation in human cells. Proc Natl Acad Sci 103: 17337–17342. 10.1073/pnas.0607015103 17085592 PMC1859931

[RNA078707CHUC25] Liu J, Carmell MA, Rivas FV, Marsden CG, Thomson JM, Song J, Hammond SM, Joshua-Tor L, Hannon GJ. 2004. Argonaute2 is the catalytic engine of mammalian RNAi. Science 305: 1437–1441. 10.1126/science.1102513 15284456

[RNA078707CHUC26] Liu J, Hu J, Corey DR. 2012. Expanding the action of duplex RNAs into the nucleus: redirecting alternative splicing. Nucleic Acids Res 40: 1240–1250. 10.1093/nar/gkr780 21948593 PMC3273794

[RNA078707CHUC27] Liu J, Liu Z, Corey DR. 2018. The requirement for GW182 scaffolding protein depends on whether argonaute is mediating translation, transcription, or splicing. Biochemistry 57: 5247–5256. 10.1021/acs.biochem.8b00602 30086238 PMC6124307

[RNA078707CHUC28] Liu Z, Johnston ST, Zhang Z, Corey DR. 2019. Expression of TNRC6 (GW182) proteins is not necessary for gene silencing by fully complementary RNA duplexes. Nucleic Acid Ther 29: 323–334. 10.1089/nat.2019.0815 31670606 PMC6885777

[RNA078707CHUC29] Lovci MT, Ghanem D, Marr H, Gee S, Parra M, Liang TY, Stark TJ, Gehman LT, Hoon S, Massirer KB, 2013. Rbfox proteins regulate alternative mRNA splicing through evolutionarily conserved RNA bridges. Nat Struct Mol Biol 20: 1434–1442. 10.1038/nsmb.2699 24213538 PMC3918504

[RNA078707CHUC30] Matsui M, Sakurai F, Elbashir S, Foster DJ, Manoharan M, Corey DR. 2010. Activation of LDL receptor expression bysmall RNAs complementary to a noncoding transcript that overlaps the LDLR promoter. Chem Biol 17: 1344–1355. 10.1016/j.chembiol.2010.10.009 21168770 PMC3071588

[RNA078707CHUC31] Matsui M, Chu Y, Zhang H, Gagnon KT, Shaikh S, Kuchimanchi S, Manoharan M, Corey DR, Janowski BA. 2013. Promoter RNA links transcriptional regulation of inflammatory pathway genes. Nucleic Acids Res 41: 10086–10109. 10.1093/nar/gkt777 23999091 PMC3905862

[RNA078707CHUC32] Meister G. 2013. Argonaute proteins: functional insights and emerging roles. Nat Rev Genet 14: 447–459. 10.1038/nrg3462 23732335

[RNA078707CHUC33] Meister G, Lanthaler M, Patkaniowska A, Dorsett Y, Teng G, Tuschl T. 2004. Human argonaute2 mediates RNA cleavage targeted by miRNAs and siRNAs. Mol Cell 15: 185–197. 10.1016/j.molcel.2004.07.007 15260970

[RNA078707CHUC34] Michlewski G, Caceres JF. 2019. Post-transcriptional control of miRNA biogenesis. RNA 25: 1–16. 10.1261/rna.068692.118 30333195 PMC6298569

[RNA078707CHUC35] Moore MJ, Scheel TKH, Luna JM, Park CY, Fak JJ, Nishiuchi E, Rice CM, Darnell RB. 2015. miRNA-target chimeras reveal miRNA 3′-end pairing as a major determinant of Argonaute target specificity. Nat Commun 6: 8863. 10.1038/ncomms9863 26602609 PMC4674787

[RNA078707CHUC36] Morris KV, Chan S W-L, Jacobsen SE, Looney DJ. 2004. Small interfering RNA-induced transcriptional gene silencing in human cells. Science 305: 1289–1292. 10.1126/science.1101372 15297624

[RNA078707CHUC37] Park MS, Sim G, Kehling AC. 2020. Human Argonaute2 and Argonaute3 are catalytically activated by different lengths of guide RNA. Proc Natl Acad Sci 117: 28576–28578. 10.1073/pnas.2015026117 33122430 PMC7682322

[RNA078707CHUC38] Petri S, Dueck A, Lemann G, Putz N, Rudel S, Kremmer E, Meister G. 2011. Increased siRNA duplex stability correlates with reduced off-target and elevated on-target effects. RNA 17: 737–749. 10.1261/rna.2348111 21367974 PMC3062184

[RNA078707CHUC39] Place RF, Li LC, Pookot D, Noonan EJ, Dahiya R. 2008. MicroRNA-373 induces expression of genes with complementary promoter sequences. Proc Natl Acad Sci 108: 1608–1613. 10.1073/pnas.0707594105 PMC2234192 18227514

[RNA078707CHUC40] Portnoy V, Lin SHS, Li KH, Burlingame A, Hu Z-H, Li H, Li LC. 2016. saRNA-guided Ago2 targets the RITA complex to promoters to stimulate transcription. Cell Res 26: 320–335. 10.1038/cr.2016.22 26902284 PMC4783471

[RNA078707CHUC41] Robb GB, Brown KM, Khurana J, Rana TM. 2005. Specific and potent RNAi in the nucleus of human cells. Nat Struct Mol Biol 12: 133–137. 10.1038/nsmb886 15643423

[RNA078707CHUC42] Sarshad AA, Juan AH, Muler AIC, Anastasakis DG, Wang X, Genzor P, Feng X, Tsai P-F, Sun H-W, Haase AD, 2018. Argonaute-miRNA complexes slicing target mRNAs in the nucleus of mammalian stem cells. Mol Cell 71: 1040–1050. 10.1016/j.molcel.2018.07.020 30146314 PMC6690358

[RNA078707CHUC43] Stalder L, Heusermann W, Sokol L, Trojer D, Wirz J, Hean J, Fritzsche A, Aeschimann F, Pfanzagl V, Basselet P, 2013. The rough endoplasmatic reticulum is a central nucleation site of siRNA-mediated RNA silencing. EMBO J 32: 1115–1127. 10.1038/emboj.2013.52 23511973 PMC3630355

[RNA078707CHUC44] Su S, Trombly MI, Chen J, Wang X. 2009. Essential and overlapping functions for mammalian argonautes in microRNA silencing. Genes Dev 23: 304–317. 10.1101/gad.1749809 19174539 PMC2648544

[RNA078707CHUC45] Treiber T, Treiber N, Meister G. 2019. Regulation of microRNA biogenesis and its crosstalk with other cellular pathways. Nat Rev Mol Cell Biol 20: 5–20. 10.1038/s41580-018-0059-1 30728477

[RNA078707CHUC46] Van Eijl RAPM, van den Brand T, Nguyen LN, Mulder KW. 2017. Reactivity of human AGO2 monoclonal antibody 11A9 with the SWI/SNF complex: a case study for rigorously defining antibody selectivity. Sci Rep 7: 7278. 10.1038/s41598-017-07539-4 28779093 PMC5544689

[RNA078707CHUC47] Van Nostrand EL, Pratt GA, Shiskin AA, Gelboin-Burhart C, Fang MY, Sundararaman B, Blue SM, Nguen TB, Surka C. 2016. Robust transcriptome-wide discovery of RNA-binding protein sites with enhanced CLIP (eCLIP). Nat Methods 13: 508–514. 10.1038/nmeth.3810 27018577 PMC4887338

[RNA078707CHUC48] Yang YC, Di C, Hu B, Zhou M, Liu Y, Song N, Li Y, Umetsu J, Lu ZJ. 2015. CLIPdb: a CLIP-seq database for protein-RNA interactions. BMC Genomics 16: 51. 10.1186/s12864-015-1273-2 25652745 PMC4326514

[RNA078707CHUC49] Younger ST, Corey DR. 2011. Transcriptional gene silencing in mammalian cells by miRNA mimics that target gene promoters. Nucleic Acids Res 39: 5682–5691. 10.1093/nar/gkr155 21427083 PMC3141263

[RNA078707CHUC50] Zeng Y, Cullen BR. 2002. RNA interference in human cells is restricted to the cytoplasm. RNA 8: 855–860. 10.1017/S1355838202020071 12166640 PMC1370302

[RNA078707CHUC51] Zhang X, Li H, Burnett JC, Rossi JJ. 2014. The role of antisense long noncoding RNA in small RNA triggered gene activation. RNA 20: 1916–1928. 10.1261/rna.043968.113 25344398 PMC4238356

[RNA078707CHUC52] Zhu Y, Xu G, Yang YT, Xu Z, Chen X, Shi B, Xie D, Lu ZJ, Wang P. 2019. POSTAR2: deciphering the post-transcriptional regulatory logics. Nucleic Acids Res 47: D203–D211. 10.1093/nar/gky830 30239819 PMC6323971

