# Supplementary material for: Argonaute binding within human nuclear RNA and its impact on alternative splicing
Source: RNA. 2021 Sep;27(9):991–1003. doi: 10.1261/rna.078707.121 (PMC8370746; doi:10.1261/rna.078707.121)
Supplement: Supplemental Material [file supp_27_9_991__DC1.html]

Argonaute binding within human nuclear RNA and its impact on alternative splicing — Supplemental Material 

# Argonaute binding within human nuclear RNA and its impact on alternative splicing

## Supplemental Material

- Supplemental\_Figures\_and\_Tables.pdf
