# Supplementary material for: Argonaute binding within human nuclear RNA and its impact on alternative splicing
Source: RNA. 2021 Sep;27(9):991–1003. doi: 10.1261/rna.078707.121 (PMC8370746; doi:10.1261/rna.078707.121)
Supplement: Supplemental Material [file supp_078707.121_Supplemental_Figures_and_Tables.pdf]

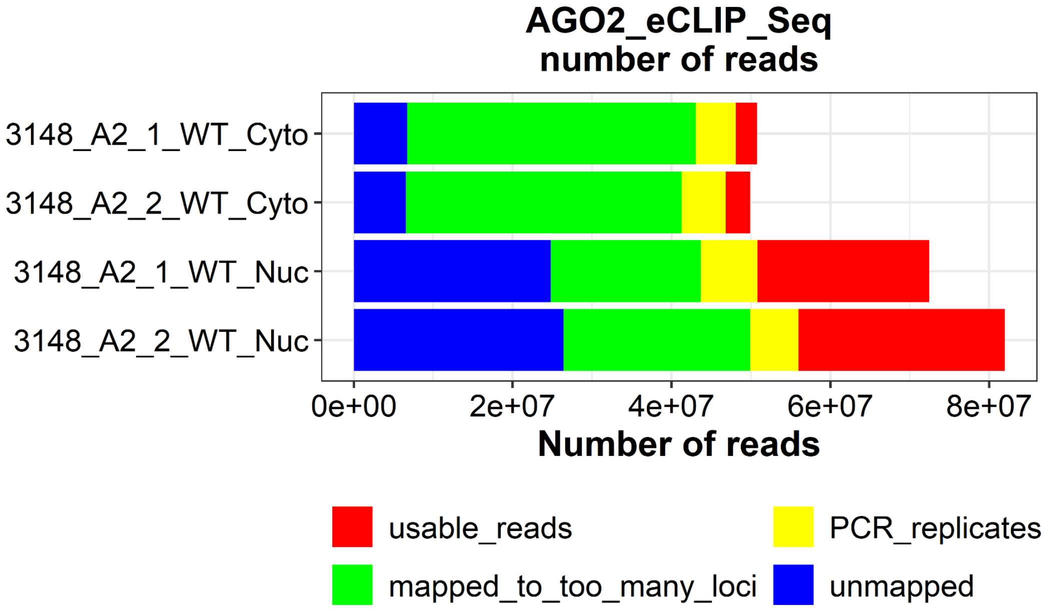

Supplemental Figure S1. Usable reads identified by anti-AGO2 eCLIP-seq in nuclear and cytoplasmic samples. Sequencing reads distribution, showing the total number of usable reads in two sets of duplicate samples. Samples were prepared from separate experimental replicates.

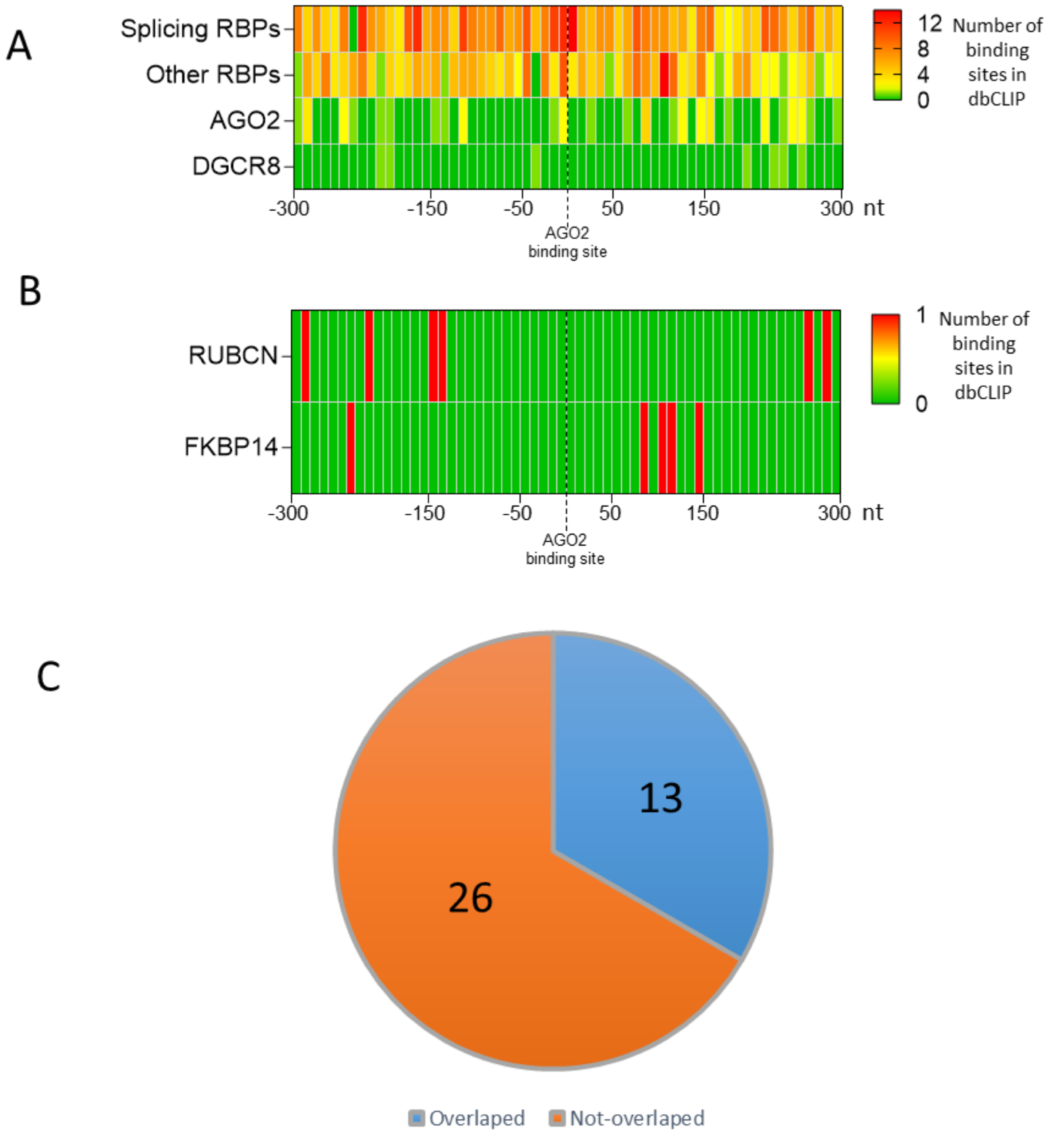

Supplementary Figure S2. Analysis of the thirty nine alternative splicing events associated with AGO2 binding (Related to Figure 4CE) and overlap with RNA binding sites from dbCLIP. (A) Distribution of splicing factors and other RBPs surrounding AGO2-binding site. (B) Distribution of RNA binding proteins near AGO2-binding site in *RUBCN* and *FKBP14*. (C) Number of alternative splicing associated AGO2-binding sites overlapped with splicing factors binding site (max 10 nt distance). We note that CLIP data was obtained using different CLIP techniques, in different cell lines, and at different sensitivities and should be interpreted with caution.

A ***KIF21A***

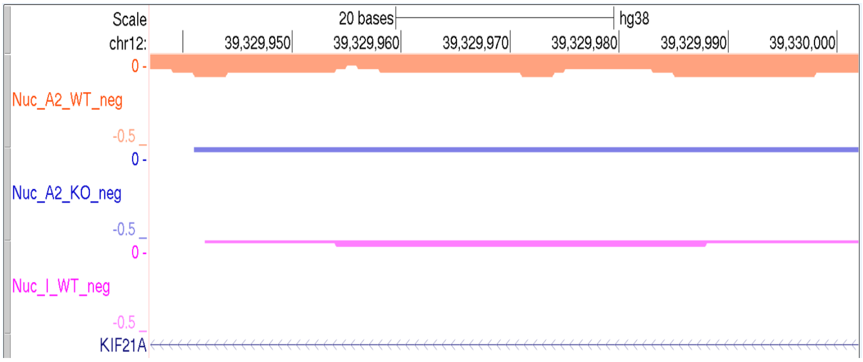

B

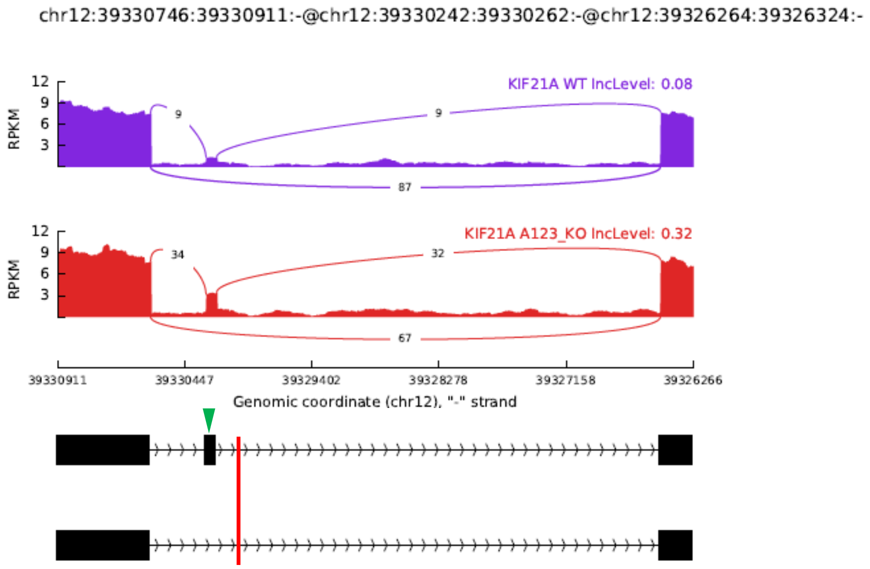

C

Expression Change in *AGO* KO cell lines by RNAseq (Relative Fold)				Expression Change in *AGO123*$^{-/-}$ cell line by qPCR (Relative Fold)
A1$^{-/-}$	A2$^{-/-}$	A1/2$^{-/-}$	A1/2/3$^{-/-}$	A1/2/3$^{-/-}$
1.11	1.08	0.88	1.14	1.08

Supplemental Figure S3A. Splicing change gene candidate in *AGO1/2/3* KO cells with AGO2 binding cluster: *KIF21A*. A. AGO2 binding clusters within *KIF21A* identified by AGO2 eCLIP-seq. Orange: Wild type cells. Blue: *AGO2* knockout cells. Pink: Wild type input control. All clusters were located in skipped exon events nearby within intron. B. Sashimi plot for significant skipped exon event by RNA-seq analysis. Purple: Wild type cells. Red: *AGO1/2/3* knockout cells. Green arrowhead: excluded/included exon. Red vertical line: location of AGO2 binding cluster. For inclusion in analysis we required peaks to possess a p value <0.05 and a >4-fold enrichment in read number for wild-type verse *AGO2* knockouts. C. Expression change by RNA Seq and qPCR in AGO KO cell lines.

PPIP5K2

A

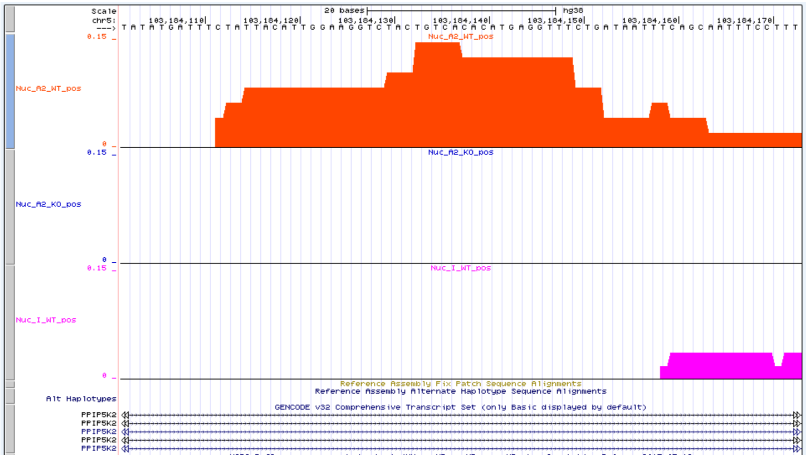

B

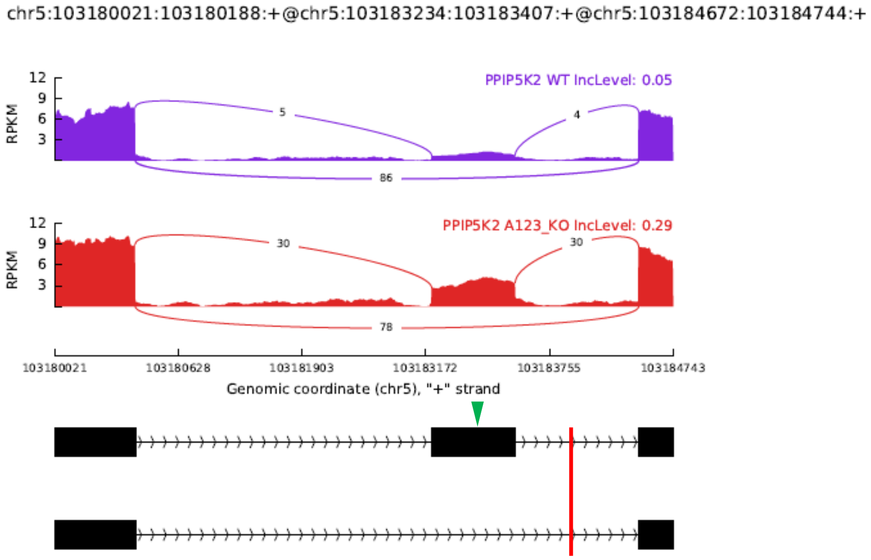

C

Expression Change in *AGO* KO cell lines by RNAseq (Relative Fold)				Expression Change in *AGO123*$^{-/-}$ cell line by qPCR (Relative Fold)
A1$^{-/-}$	A2$^{-/-}$	A1/2$^{-/-}$	A1/2/3$^{-/-}$	A1/2/3$^{-/-}$
0.91	1.33	0.90	1.26	1.40

Supplemental Figure S3C. Splicing change gene candidate in *AGO1/2/3* KO cells with AGO2 binding Bcluster: *PPIP5K2*. A. AGO2 binding clusters within *PPIP5K2* identified by AGO2 eCLIP-seq. Red: Wild type cells. Navy: *AGO2* knockout cells. Pink: Wild type input control. All clusters were located in skipped exon events nearby within intron. B. Sashimi plot for significant skipped exon event by RNA-seq analysis. Purple: Wild type cells. Red: *AGO1/2/3* knockout cells. Green arrowhead: excluded/included exon. Red vertical line: location of AGO2 binding cluster. For inclusion in analysis we required peaks to possess a *p* value <0.05 and a >4-fold enrichment in read number for wild-type verse *AGO2* knockouts. C. Expression change by RNA Seq and qPCR in AGO KO cell lines.

A

PHLDB1

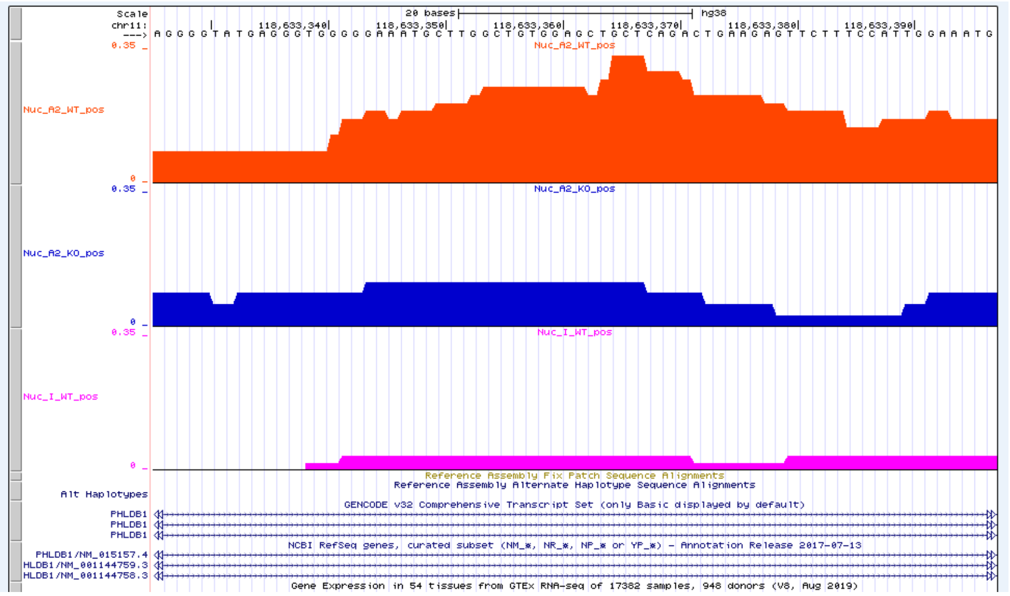

B

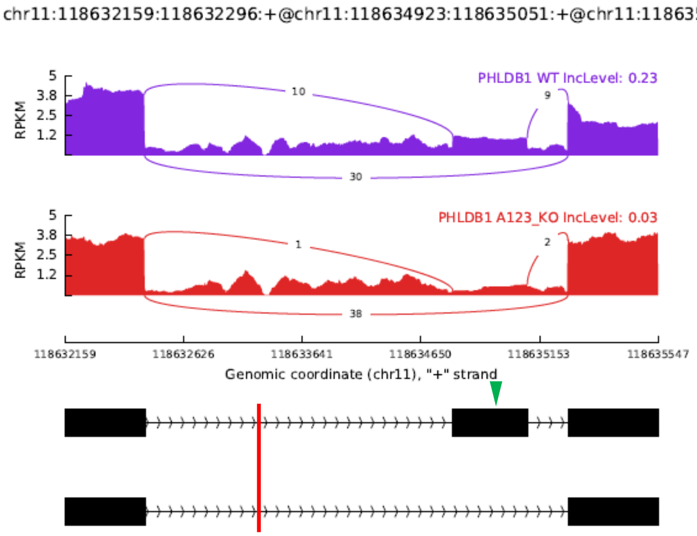

C

Expression Change in *AGO* KO cell lines by RNAseq (Relative Fold)				Expression Change in *AGO123*$^{-/-}$ cell line by qPCR (Relative Fold)
A1$^{-/-}$	A2$^{-/-}$	A1/2$^{-/-}$	A1/2/3$^{-/-}$	A1/2/3$^{-/-}$
0.98	0.96	1.07	1.14	1.22

Supplemental Figure S3C. Splicing change gene candidate in AGO1/2/3 KO cells with AGO2 binding cluster: *PHLDB1*. A. AGO2 binding clusters within *PHLDB1* identified by AGO2 eCLIP-seq. Red: Wild type cells. Navy: *AGO2* knockout cells. Pink: Wild type input control. All clusters were located in skipped exon events nearby within intron. B. Sashimi plot for significant skipped exon event by RNA-seq analysis. Purple: Wild type cells. Red: *AGO1/2/3* knockout cells. Green arrowhead: excluded/included exon. Red vertical line: location of AGO2 binding cluster. For inclusion in analysis we required peaks to possess a *p* value <0.05 and a >4-fold enrichment in read number for wild-type verse *AGO2* knockouts. C. Expression change by RNA Seq and qPCR in AGO KO cell lines.

FKBP14

A

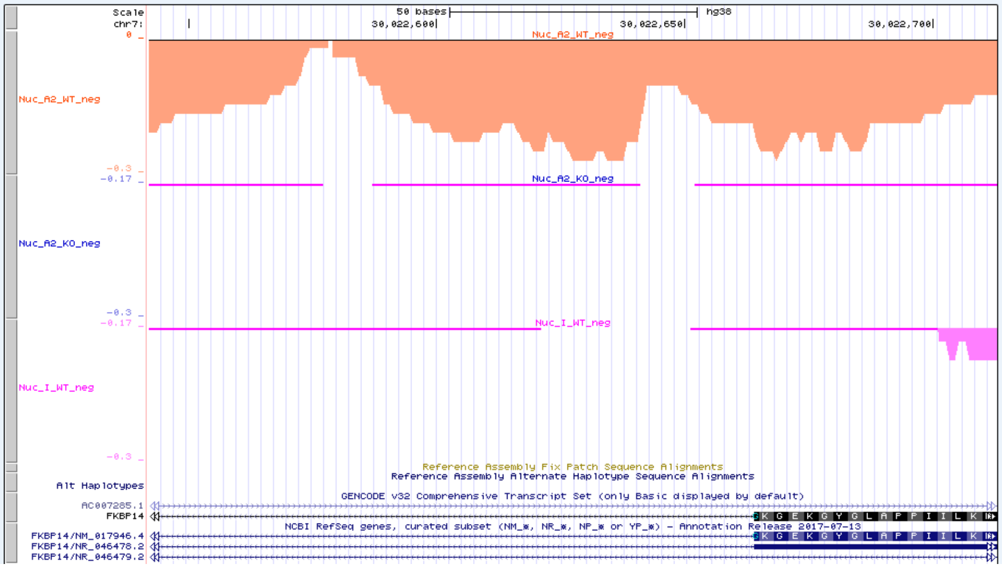

B

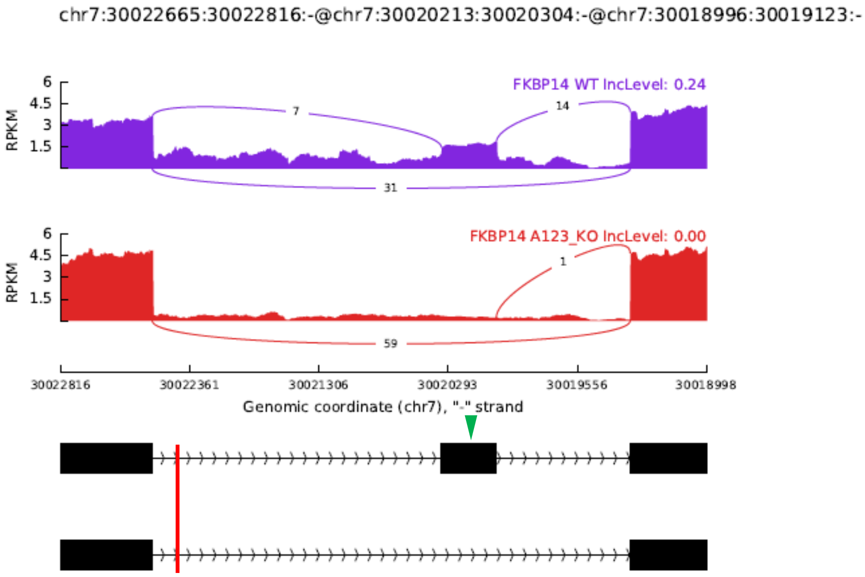

C

Expression Change in *AGO* KO cell lines by RNAseq (Relative Fold)				Expression Change in $AGO123^{-/-}$ cell line by qPCR (Relative Fold)
$A1^{-/-}$	$A2^{-/-}$	$A1/2^{-/-}$	$A1/2/3^{-/-}$	$A1/2/3^{-/-}$
0.88	0.88	0.74	0.76	1.27

Supplemental Figure S3D. Splicing change gene candidate in *AGO1/2/3* KO cells with AGO2 binding cluster: *FKBP14*. A. AGO2 binding clusters within *FKBP14* identified by AGO2 eCLIP-seq. Orange: Wild type cells. Blue: *AGO2* knockout cells. Pink: Wild type input control. All clusters were located in skipped exon events nearby within intron. B. Sashimi plot for significant skipped exon event by RNA-seq analysis. Purple: Wild type cells. Red: *AGO1/2/3* knockout cells. Green arrowhead: excluded/included exon. Red vertical line: location of AGO2 binding cluster. For inclusion in analysis we required peaks to possess a p value <0.05 and a >4-fold enrichment in read number for wild-type verse *AGO2* knockouts. C. Expression change by RNA Seq and qPCR in AGO KO cell lines.

A

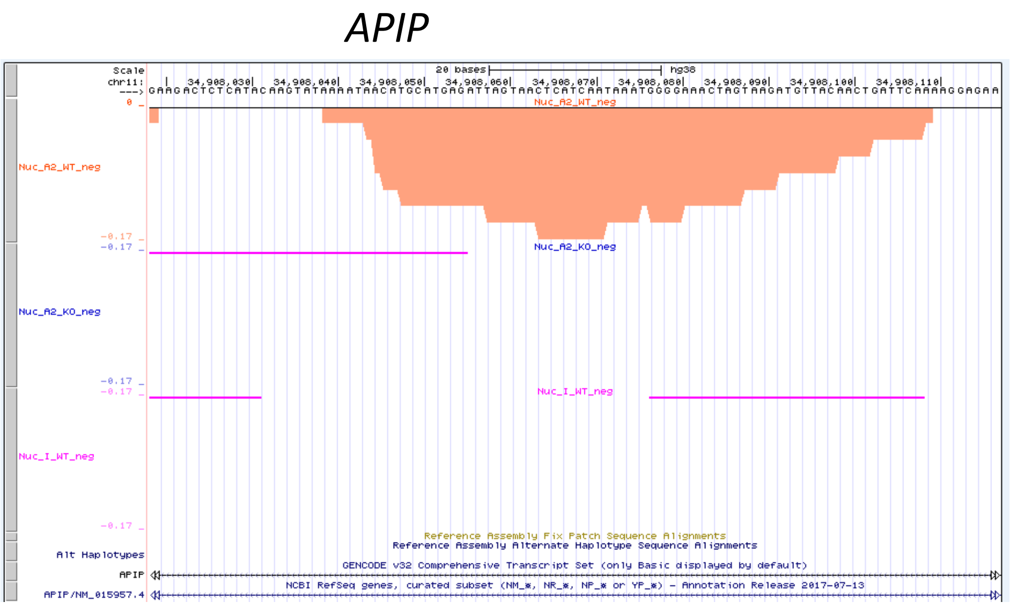

B

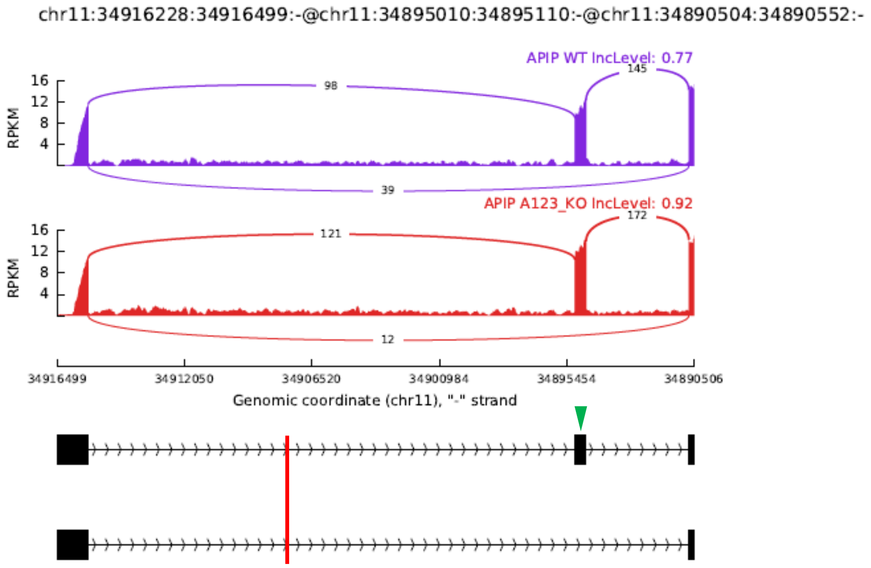

C

Expression Change in *AGO* KO cell lines by RNAseq (Relative Fold)				Expression Change in AGO123$^{-/-}$ cell line by qPCR (Relative Fold)
A1$^{-/-}$	A2$^{-/-}$	A1/2$^{-/-}$	A1/2/3$^{-/-}$	A1/2/3$^{-/-}$
0.87	1.04	0.94	0.91	0.73

Supplemental Figure S3E. Splicing change gene candidate in *AGO1/2/3* KO cells with AGO2 binding cluster: *APIP*. A. AGO2 binding clusters within *APIP* identified by AGO2 eCLIP-seq. Orange: Wild type cells. Blue: *AGO2* knockout cells. Pink: Wild type input control. All clusters were located in skipped exon events nearby within intron. B. Sashimi plot for significant skipped exon event by RNA-seq analysis. Purple: Wild type cells. Red: *AGO1/2/3* knockout cells. Green arrowhead: excluded/included exon. Red vertical line: location of AGO2 binding cluster. For inclusion in analysis we required peaks to possess a *p* value <0.05 and a >4-fold enrichment in read number for wild-type verse *AGO2* knockouts. C. Expression change by RNA Seq and qPCR in AGO KO cell lines.

A

RUBCN

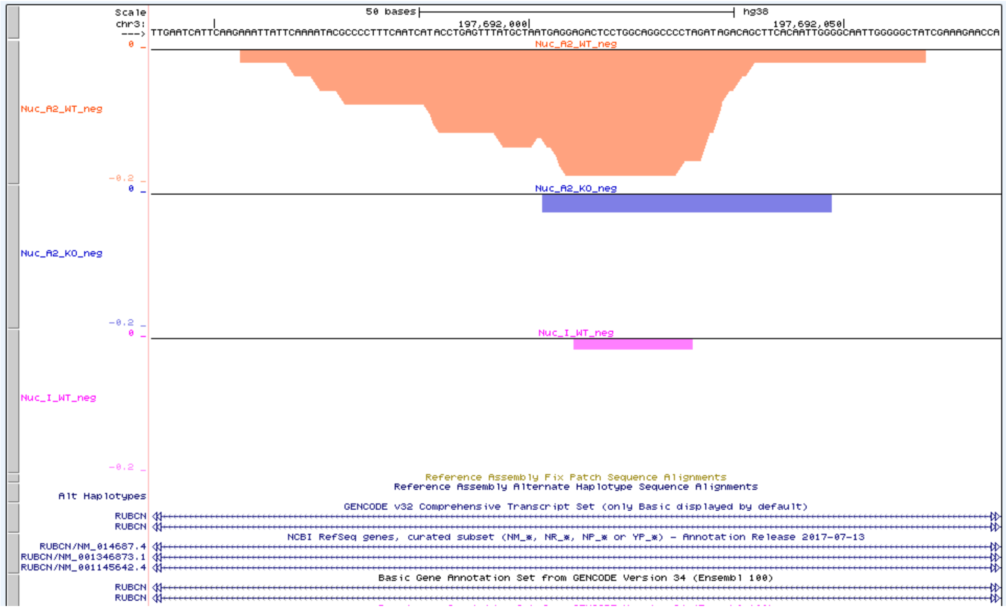

B

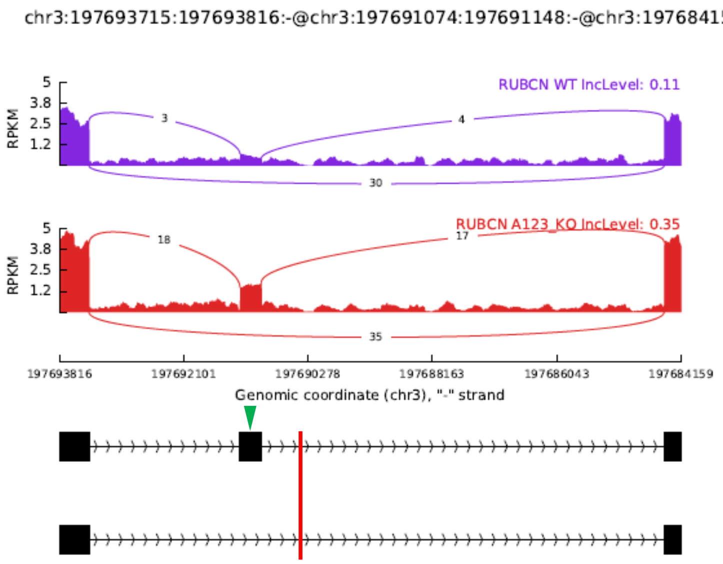

C

Expression Change in *AGO* KO cell lines by RNAseq (Relative Fold)				Expression Change in *AGO123*$^{-/-}$ cell line by qPCR (Relative Fold)
A1$^{-/-}$	A2$^{-/-}$	A1/2$^{-/-}$	A1/2/3$^{-/-}$	A1/2/3$^{-/-}$
0.90	0.98	0.97	1.21	1.02

Supplemental Figure S3F. Splicing change gene candidate in *AGO1/2/3* KO cells with AGO2 binding cluster: *RUBCN*. A. AGO2 binding clusters within *RUBCN* identified by AGO2 eCLIP-seq. Orange: Wild type cells. Blue: *AGO2* knockout cells. Pink: Wild type input control. All clusters were located in skipped exon events nearby within intron. B. Sashimi plot for significant skipped exon event by RNA-seq analysis. Purple: Wild type cells. Red: *AGO1/2/3* knockout cells. Green arrowhead: excluded/included exon. Red vertical line: location of AGO2 binding cluster. For inclusion in analysis we required peaks to possess a *p* value <0.05 and a >4-fold enrichment in read number for wild-type verse *AGO2* knockouts. C. Expression change by RNA Seq and qPCR in AGO KO cell lines.

TBC1D5

A

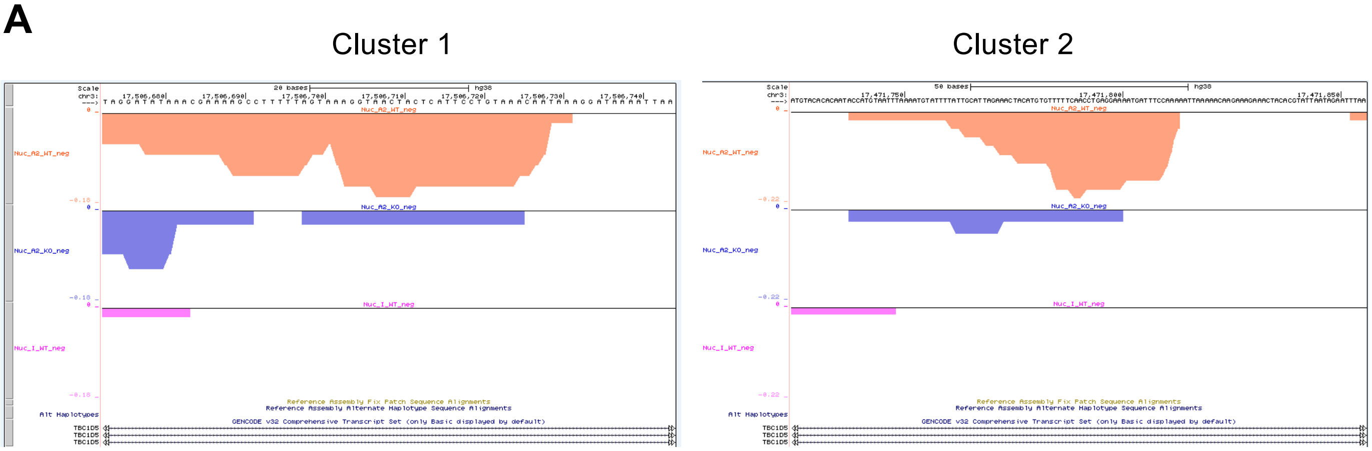

B

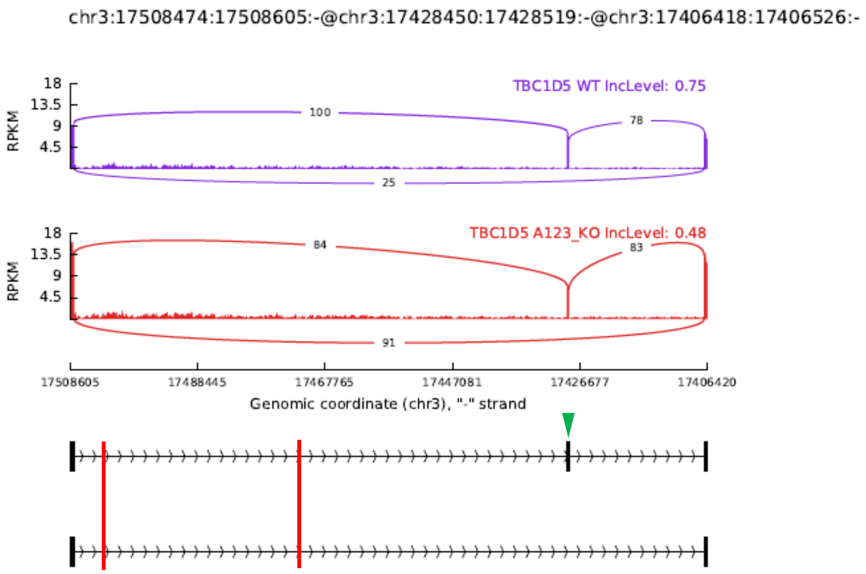

C

Expression Change in *AGO* KO cell lines by RNAseq (Relative Fold)				Expression Change in *AGO123*$^{-/-}$ cell line by qPCR (Relative Fold)
A1$^{-/-}$	A2$^{-/-}$	A1/2$^{-/-}$	A1/2/3$^{-/-}$	A1/2/3$^{-/-}$
1.00	1.27	1.44	1.53	0.98

Supplemental Figure S3G. Splicing change gene candidate in *AGO1/2/3* KO cells with AGO2 binding cluster: *TBC1D5*. A. AGO2 binding clusters within *TBC1D5* identified by AGO2 eCLIP-seq. Orange: Wild type cells. Blue: *AGO2* knockout cells. Pink: Wild type input control. All clusters were located in skipped exon events nearby within intron. B. Sashimi plot for significant skipped exon event by RNA-seq analysis. Purple: Wild type cells. Red: *AGO1/2/3* knockout cells. Green arrowhead: excluded/included exon. Red vertical line: location of AGO2 binding cluster. For inclusion in analysis we required peaks to possess a *p* value <0.05 and a >4-fold enrichment in read number for wild-type verse *AGO2* knockouts. C. Expression change by RNA Seq and qPCR in AGO KO cell lines.

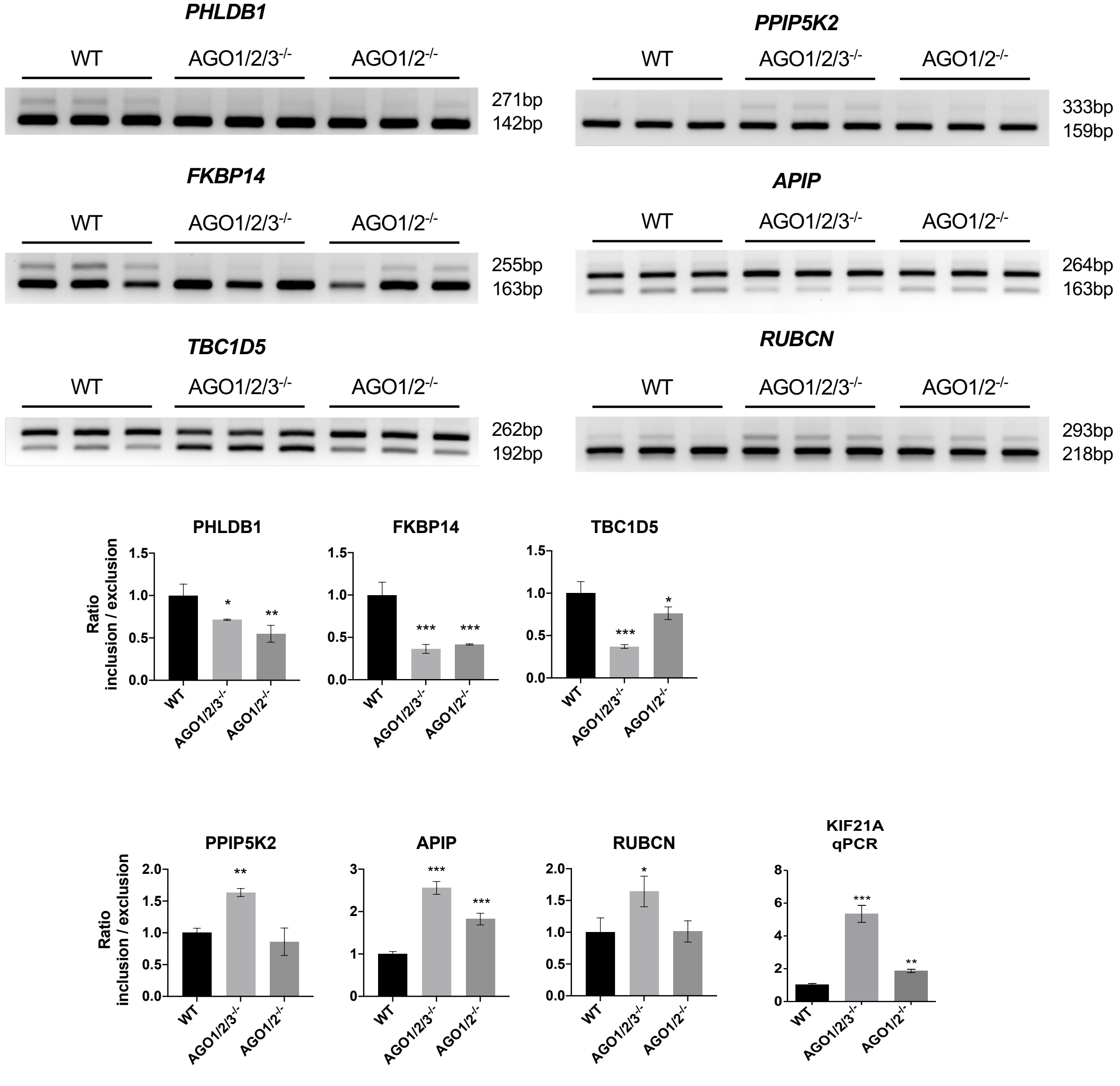

Supplemental Figure S4, related to Figure 5. Semiquantitative PCR validation of skipped exon events in *AGO1/2* KO and *AGO1/2/3* KO cells by different PCR primer sets. Splicing analysis for *KIF21A* was done by QPCR by different PCR primer sets. Error bars represent standard deviation (SD). *P < 0.05; **P < 0.01; ***P < 0.001 compared with WT by one-way ANOVA and Dunnett's multiple comparisons test.

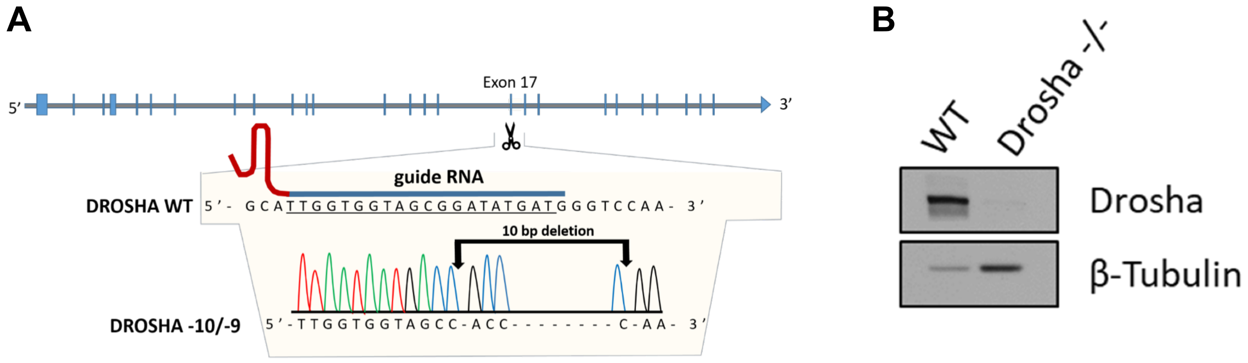

Supplemental Figure S5. Characgterization of *DROSHA* KO cells. (A) Location of guide RNA and deletions from CRISPR/Cas9-derived HCT116 *DROSHA* knockout cell line. (B) Western blot validating the *DROSHA* -10/-9 knockout cell line.

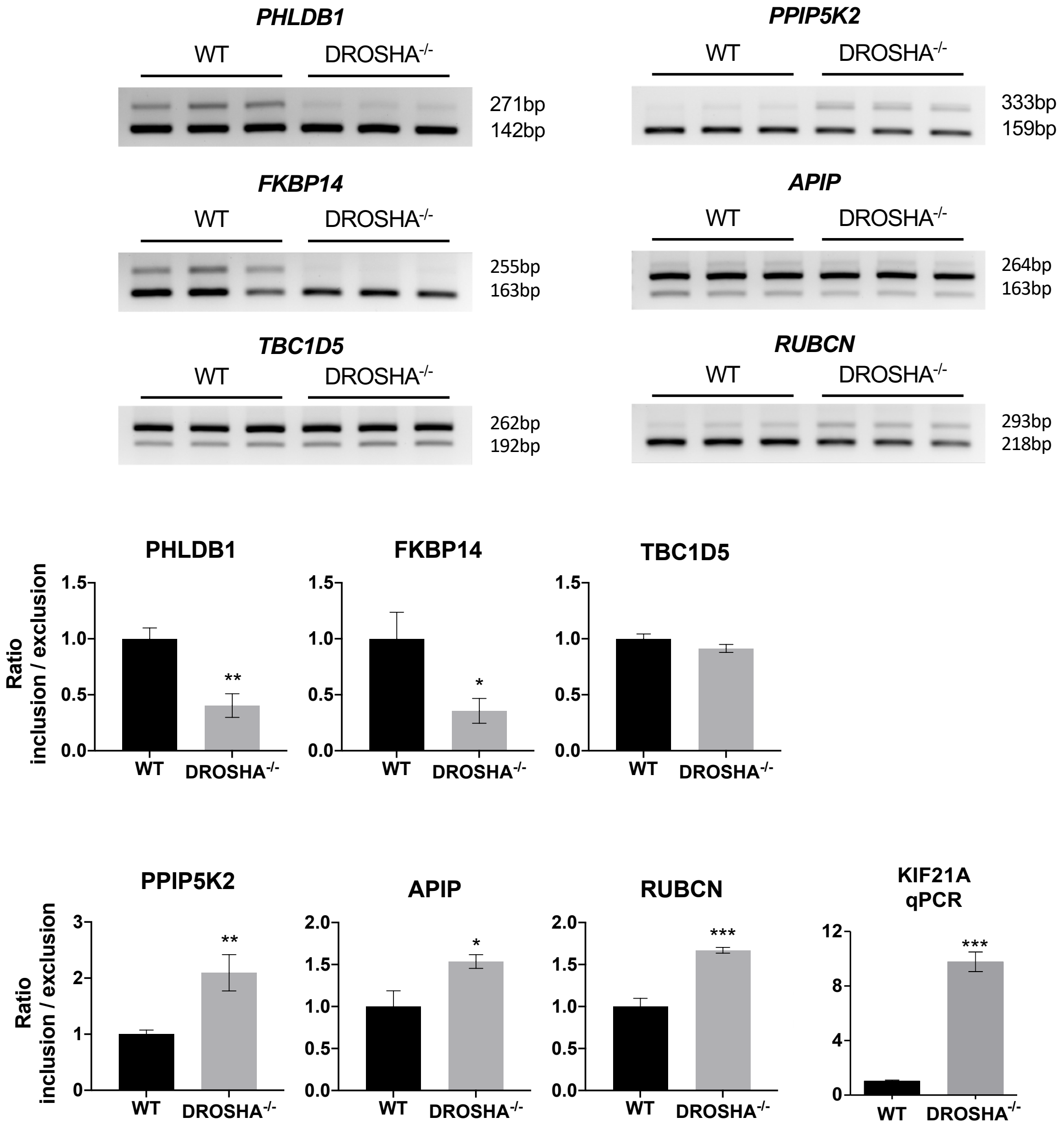

Supplemental Figure S6, related to Figure 6. Semiquantitative PCR validation of skipped exon events in *DROSHA* knockout cells by different PCR primer sets. Splicing analysis for *KIF21A* in *DROSHA* knockout cells was done by QPCR by different PCR primer sets. Error bars represent standard deviation (SD). *P < 0.05; **P < 0.01; ***P < 0.001 compared with WT by t-test.

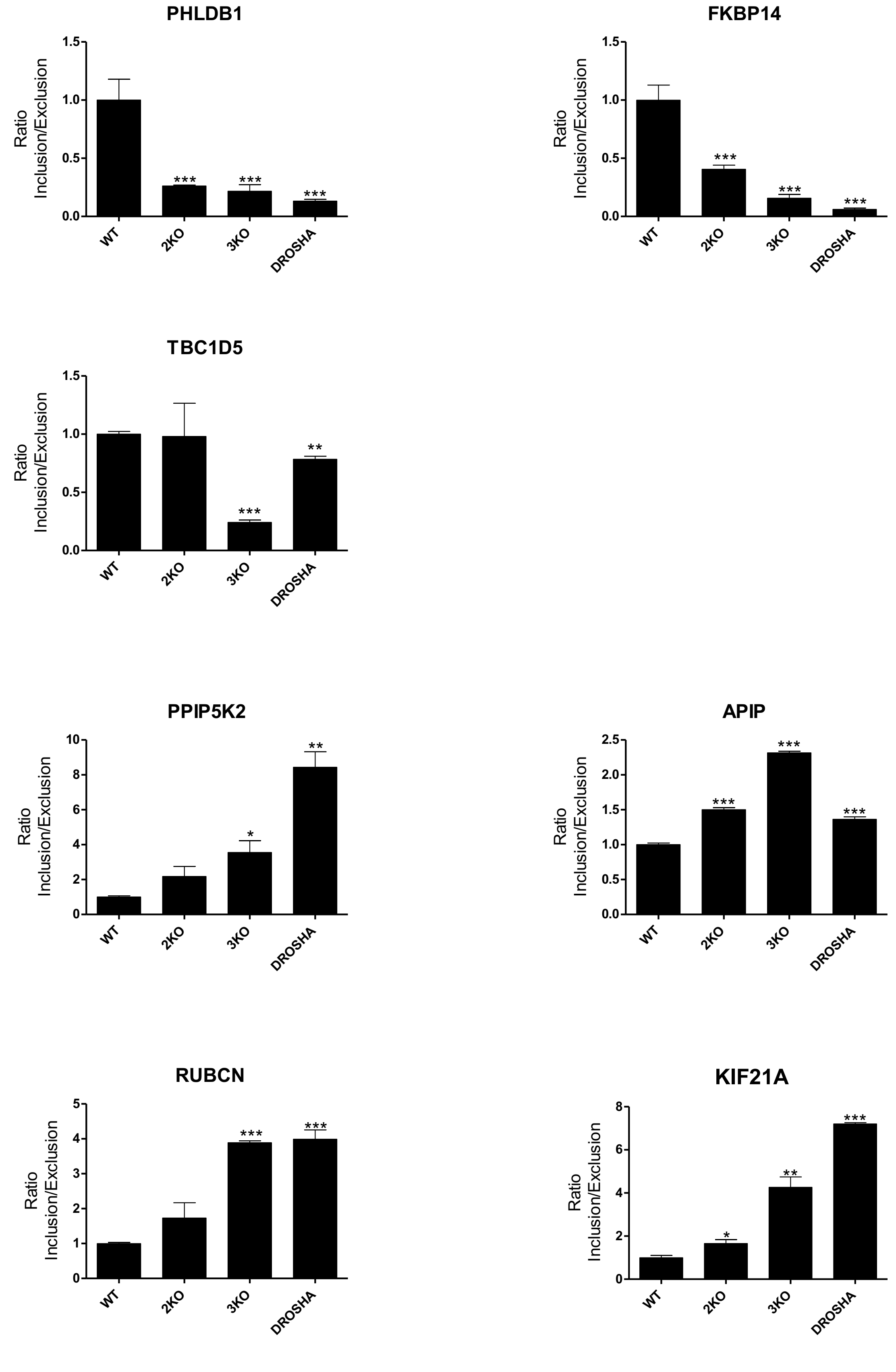

Supplemental Figure S7, related to Figures 5 and 6. Comparing skipped exon events in AGO and DROSHO knockout cells. Semiquantitative PCR validation of skipped exon events in WT, *AGO1/2* knockout, *AGO1/2/3* knockout and *DROSHA* knockout cells by different PCR primer sets. Error bars represent standard deviation (SD). *P < 0.05; **P < 0.01; ***P < 0.001 compared with WT by t-test.

Supplementary Table S1. miRNA mimic sequence

Name	strand	Sequence
miR-28-5p	3p-full	5’- CAAUAGACUGUGAGCUCCUU -3’
	5p	5’- pAAGGAGCUCACAGUCUAUUGAG -3’
miR-151a-5p	3p-full	5’- UAGACUGUGAGCUCCUCGA -3’
	5p	5’- pUCGAGGAGCUCACAGUCUAGU -3’
miR-769-5p	3p-full	5’- CUCAGAACCCAGAGGUCUCA -3’
	5p	5’- pUGAGACCUCUGGGUUCUGAGCU -3’
miR-20a-5p	3p-full	5’- ACCUGCACUAUAAGCACUUUA -3’
	5p	5’- pUAAAGUGCUUAUAGUGCAGGUAG -3’
miR-149-5p	3p-full	5’- GAGUGAAGACACGGAGCCAGA -3’
	5p	5’- pUCUGGCUCCGUGUCUUCACUCCC -3’

Supplementary Table S2. PCR primers for gel electrophoresis and qPCR analysis

Application	Name	Sequence (5'-3')
Agarose gel electrophoresis	PPIP5K2_E25_SE_F1	GATAATGATGATGAACCACATACTTC
	PPIP5K2_E25_SE_F2	AGAGATGAAGTTGATCGAGCTGTG
	PPIP5K2_E27_SE_R1	CTACAGTAGGATTCTGCTTCTGTTCC
	PPIP5K2_E27_SE_R2	CACAAGAGTTCTTGGTGTTCTCAGG
	APIP_E1_SE_F1	GCTCGGGAGGGAGACTGTTGTTC
	APIP_E3_SE_R1	CTGAATTCGTTCCTTTTGCACTCC
	APIP_5UTR_SE_F2	AAAGCCGTGCGGAGATTGGAGG
	APIP_E3/4_SE_R2	CATGTCTTCAGGCTGAATTCGTTC
	RUBCN_E12_SE_F1	AGTTCAGCTCACGTGATTCGGCAC
	RUBCN_E14_SE_R1	AAGGATTTGCTGCTTGAGGCTGTG
	RUBCN_E11_SE_F2	TACCAGGAGGCTGAGCACGGAAGC
	RUBCN_E15_SE_R2	TCAGCAGACGTGGAGTGCAGGAAG
	PHLDB1_E9_SE_F2	AGAAGGAGCAGAAGGCAGTGGATC
	PHLDB1_E9_SE_F1	AGAAGCTGGTGGCCTTGGAGACAG
	PHLDB1_E10_SE_R2	TCCAACTGCTGGAACTCCAAGTC
	FKBP14_E2_SE_F1	TGGTCAGCCCATTTGGTTTACC
	FKBP14_E2_SE_F2	GCTCATCATTCCTCCTGCTCTGG
	FKBP14_E4_SE_R1	TGGAATGATTCATGGGATCTTGGTC
	FKBP14_E4_SE_R2	AGAGAGTTTCCAGTCATCATTAAGATCC
	TBC1D5_E3_F1	AGTTACTTTTGGTGACGCTGTCC
	TBC1D5_E3_F2	ACTAGACATCCTCTGCAGCCAGAAG
	TBC1D5_E5_R1	CTGCTTCTCAGCTGCCCATTAATC
	TBC1D5_E5/6_R2	GAAATAGCTTCCAGCAAATGCTGC
	KIF21A_E22_F1	AGATGCTTTACTAGGCCATGC
	KIF21A_E25_R1	ATGAGATCTGAAGACAGCGTG
QPCR	FKBP14_E3_SE_F3	AGCGCATGTCACATCTCTGTCTAG
	FKBP14_E2/4_SE_F4	GGAAAAGAAGGAAAAGGTAAAATTCC
	FKBP14_E3_SE_R3	AGATCTAGACAGAGATGTGACATGC
	FKBP14_E2/4_SE_R4	CTTTCTGGGGGAATTTTACCTTTTCC
	RUBCN_E13/E14_SE_F4	GGCCTCGATGTTCTCAGATGCTGA
	RUBCN_E12/14_SE_F5	GAATTTGAAATCCAAGATGCTGACA
	RUBCN_E13/E14_SE_R4	CCTTCTGATGTCAGCATCTGAGAAC
	RUBCN_E12/14_SE_R5	CCTTCTGATGTCAGCATCTTGGATT
	KIF21A_IN_E22-23_F3	ACAAGATCTAGATAGCGTACCATTAG
	KIF21A_IN_E24_R3	TCTGATCCTGGGCTGTTTAAAG
	KIF21A_IN_E22_F4	CAGAAATAACCAGTGCTACCCA
	KIF21A _IN_E23-24_R4	CCTCTACATTTTCTAATGGTACGC
	KIF21A_IN_E22/24_F9	CTAGGCCATGCTTTACAAGAAAATG
	KIF21A_EX_E25_R9	ATGAGATCTGAAGACAGCGTG
	KIF21A_EX_E22/24_F11	CTAGGCCATGCTTTACAAGAA
	KIF21A_EX_E26_R11	CTCCTTCGGGCCTTGTTCTT

Supplementary Table S3. Oligonucleotides used in eCLIP library preparation

**Oligonucleotides for eCLIP**		
**Oligonucleotide name**	**Description**	**Sequence (5' to 3')**
RNA_A01	Library preparation	/5phos/rArUrUrGrCrUrUrArGrArUrCrGrGrArArGrArGrCrGrUrCrGrUrGrUrArG/3SpC3/
RNA_B06	Library preparation	/5phos/rArCrArArGrCrCrArGrArUrCrGrGrArArGrArGrCrGrUrCrGrUrGrUrArG/3SpC3/
RNA_C01	Library preparation	/5phos/rArArCrUrUrGrUrArGrArUrCrGrGrArArGrArGrCrGrUrCrGrUrGrUrArG/3SpC3/
RNA_D08	Library preparation	/5phos/rArGrGrArCrCrArArGrArUrCrGrGrArArGrArGrCrGrUrCrGrUrGrUrArG/3SpC3/
RNA_X1A	Library preparation	/5Phos/rArUrArUrArGrG rNrNrNrNrN rArGrArUrCrGrGrArArGrArGrCrGrUrCrGrUrGrUrArG/3SpC3/
RNA_X1B	Library preparation	/5Phos/rArArUrArGrCrA rNrNrNrNrN rArGrArUrCrGrGrArArGrArGrCrGrUrCrGrUrGrUrArG/3SpC3/
RNA_X2A	Library preparation	/5Phos/rArArGrUrArUrA rNrNrNrNrN rArGrArUrCrGrGrArArGrArGrCrGrUrCrGrUrGrUrArG/3SpC3/
RNA_X2B	Library preparation	/5Phos/rArGrArArGrArU rNrNrNrNrN rArGrArUrCrGrGrArArGrArGrCrGrUrCrGrUrGrUrArG/3SpC3/
RiL19	Library preparation	/5Phos/AGAUCGGAAGAGCGUCGUG/3SpC3/
rand103Tr3	Library preparation	/5Phos/NNNNNNNNNNAGATCGGAAGAGCACACGTCTG/3SpC3/
AR17	Library preparation	ACACGACGCTCTTCCGA
PCR_F_D501 NextSeq	Library amplification	AATGATACGGCGACCACCGAGATCTACAC**AGGCTATA**ACACTCTTTCCCTACACGACGCTCTTCCGATCT
PCR_F_D502 NextSeq	Library amplification	AATGATACGGCGACCACCGAGATCTACAC**GCCTCTAT**ACACTCTTTCCCTACACGACGCTCTTCCGATCT
PCR_F_D503 NextSeq	Library amplification	AATGATACGGCGACCACCGAGATCTACAC**AGGATAGG**ACACTCTTTCCCTACACGACGCTCTTCCGATCT
PCR_F_D504 NextSeq	Library amplification	AATGATACGGCGACCACCGAGATCTACAC**TCAGAGCC**ACACTCTTTCCCTACACGACGCTCTTCCGATCT
PCR_R_D701	Library amplification	CAAGCAGAAGACGGCATACGAGAT**CGAGTAAT**GTGACTGGAGTTCAGACGTGTGCTCTTCCGATC
PCR_R_D702	Library amplification	CAAGCAGAAGACGGCATACGAGAT**TCTCCGGA**GTGACTGGAGTTCAGACGTGTGCTCTTCCGATC
PCR_R_D703	Library amplification	CAAGCAGAAGACGGCATACGAGAT**AATGAGCG**GTGACTGGAGTTCAGACGTGTGCTCTTCCGATC
PCR_R_D704	Library amplification	CAAGCAGAAGACGGCATACGAGAT**GGAATCTC**GTGACTGGAGTTCAGACGTGTGCTCTTCCGATC

Sup. Table S4. Splicing associated proteins dysregulated by AGOs knock-down. Genes shaded in pink are down-regulated, genes shaded in green are up-regulated. Significance is defined as P<0.05.

Major splicoesome proteins

Symbol	Name	Group name	Log2 Fold Change (AGO123-/- / WT)
CASC3	CASC3 exon junction complex subunit	Exon junction complex	N.S.
EIF4A3	eukaryotic translation initiation factor 4A3	Exon junction complex	N.S.
MAGOH	mago homolog, exon junction complex subunit	Exon junction complex	N.S.
MAGOHB	mago homolog B, exon junction complex subunit	Exon junction complex	N.S.
RBM8A	RNA binding motif protein 8A	Exon junction complex	N.S.
LSM1	LSM1 homolog, mRNA degradation associated	LSm proteins	N.S.
LSM10	LSM10, U7 small nuclear RNA associated	LSm proteins	N.S.
LSM11	LSM11, U7 small nuclear RNA associated	LSm proteins	N.S.
LSM12	LSM12 homolog	LSm proteins	N.S.
LSM14A	LSM14A mRNA processing body assembly factor	LSm proteins	N.S.
LSM14B	LSM family member 14B	LSm proteins	N.S.
LSM2	LSM2 homolog, U6 small nuclear RNA and mRNA degradation associated	LSm proteins	-0.84508
LSM3	LSM3 homolog, U6 small nuclear RNA and mRNA degradation associated	LSm proteins	N.S.
LSM4	LSM4 homolog, U6 small nuclear RNA and mRNA degradation associated	LSm proteins	N.S.
LSM5	LSM5 homolog, U6 small nuclear RNA and mRNA degradation associated	LSm proteins	N.S.
LSM6	LSM6 homolog, U6 small nuclear RNA and mRNA degradation associated	LSm proteins	-0.614138
LSM7	LSM7 homolog, U6 small nuclear RNA and mRNA degradation associated	LSm proteins	N.S.
LSM8	LSM8 homolog, U6 small nuclear RNA associated	LSm proteins	N.S.
NAA38	N-alpha-acetyltransferase 38, NatC auxiliary subunit	LSm proteins	N.S.
BCAS2	BCAS2 pre-mRNA processing factor	NineTeen complex	N.S.
CDC5L	cell division cycle 5 like	NineTeen complex	N.S.
CTNNBL1	catenin beta like 1	NineTeen complex	N.S.
CWC15	CWC15 spliceosome associated protein homolog	NineTeen complex	N.S.
PLRG1	pleiotropic regulator 1	NineTeen complex	N.S.
PRPF19	pre-mRNA processing factor 19	NineTeen complex	N.S.
SF3A1	splicing factor 3a subunit 1	SF3a complex	N.S.
SF3A2	splicing factor 3a subunit 2	SF3a complex	N.S.
SF3A3	splicing factor 3a subunit 3	SF3a complex	N.S.
PHF5A	PHD finger protein 5A	SF3b complex	N.S.
SF3B1	splicing factor 3b subunit 1	SF3b complex	N.S.
SF3B2	splicing factor 3b subunit 2	SF3b complex	N.S.
SF3B3	splicing factor 3b subunit 3	SF3b complex	N.S.
SF3B4	splicing factor 3b subunit 4	SF3b complex	N.S.
SF3B5	splicing factor 3b subunit 5	SF3b complex	N.S.
SF3B6	splicing factor 3b subunit 6	SF3b complex	N.S.
SNRPB	small nuclear ribonucleoprotein polypeptides B and B1	Sm spliceosomal proteins	N.S.
SNRPD1	small nuclear ribonucleoprotein D1 polypeptide	Sm spliceosomal proteins	N.S.
SNRPD2	small nuclear ribonucleoprotein D2 polypeptide	Sm spliceosomal proteins	-0.920187
SNRPD3	small nuclear ribonucleoprotein D3 polypeptide	Sm spliceosomal proteins	N.S.
SNRPE	small nuclear ribonucleoprotein polypeptide E	Sm spliceosomal proteins	-0.744468
SNRPF	small nuclear ribonucleoprotein polypeptide F	Sm spliceosomal proteins	-0.752042
SNRPG	small nuclear ribonucleoprotein polypeptide G	Sm spliceosomal proteins	N.S.
SNRPN	small nuclear ribonucleoprotein polypeptide N	Sm spliceosomal proteins	N.S.
CCAR1	cell division cycle and apoptosis regulator 1	Spliceosomal A complex	N.S.
CHERP	calcium homeostasis endoplasmic reticulum protein	Spliceosomal A complex	N.S.
DDX46	DEAD-box helicase 46	Spliceosomal A complex	N.S.
DHX15	DEAH-box helicase 15	Spliceosomal A complex	N.S.
HNRNPA1	heterogeneous nuclear ribonucleoprotein A1	Spliceosomal A complex	N.S.
HNRNPAB	heterogeneous nuclear ribonucleoprotein A/B	Spliceosomal A complex	N.S.
PRPF40A	pre-mRNA processing factor 40 homolog A	Spliceosomal A complex	N.S.
PUF60	poly(U) binding splicing factor 60	Spliceosomal A complex	N.S.
RBM10	RNA binding motif protein 10	Spliceosomal A complex	N.S.
RBM17	RNA binding motif protein 17	Spliceosomal A complex	N.S.
RBM25	RNA binding motif protein 25	Spliceosomal A complex	N.S.
RBM5	RNA binding motif protein 5	Spliceosomal A complex	N.S.
SF1	splicing factor 1	Spliceosomal A complex	N.S.
SMNDC1	survival motor neuron domain containing 1	Spliceosomal A complex	N.S.
SUGP1	SURP and G-patch domain containing 1	Spliceosomal A complex	N.S.
THRAP3	thyroid hormone receptor associated protein 3	Spliceosomal A complex	N.S.
U2AF1	U2 small nuclear RNA auxiliary factor 1	Spliceosomal A complex	N.S.
U2AF2	U2 small nuclear RNA auxiliary factor 2	Spliceosomal A complex	N.S.
U2SURP	U2 snRNP associated SURP domain containing	Spliceosomal A complex	N.S.
AQR	aquarius intron-binding spliceosomal factor	Spliceosomal B complex	N.S.
BUD31	BUD31 homolog	Spliceosomal B complex	N.S.
CRNKL1	crooked neck pre-mRNA splicing factor 1	Spliceosomal B complex	N.S.
IK	IK cytokine	Spliceosomal B complex	N.S.
ISY1	ISY1 splicing factor homolog	Spliceosomal B complex	N.S.
MFAP1	microfibril associated protein 1	Spliceosomal B complex	N.S.
PPIE	peptidylprolyl isomerase E	Spliceosomal B complex	N.S.
PPIL1	peptidylprolyl isomerase like 1	Spliceosomal B complex	N.S.
PQBP1	polyglutamine binding protein 1	Spliceosomal B complex	N.S.
PRPF38A	pre-mRNA processing factor 38A	Spliceosomal B complex	N.S.
RBM22	RNA binding motif protein 22	Spliceosomal B complex	N.S.
SMU1	SMU1 DNA replication regulator and spliceosomal factor	Spliceosomal B complex	N.S.
SNW1	SNW domain containing 1	Spliceosomal B complex	N.S.
TFIP11	tuftelin interacting protein 11	Spliceosomal B complex	N.S.
WBP11	WW domain binding protein 11	Spliceosomal B complex	N.S.
WBP4	WW domain binding protein 4	Spliceosomal B complex	N.S.
XAB2	XPA binding protein 2	Spliceosomal B complex	N.S.
ZMAT2	zinc finger matrin-type 2	Spliceosomal B complex	N.S.
CCDC12	coiled-coil domain containing 12	Spliceosomal Bact complex	N.S.
CDC40	cell division cycle 40	Spliceosomal Bact complex	N.S.
CWC22	CWC22 spliceosome associated protein homolog	Spliceosomal Bact complex	N.S.
CWC25	CWC25 spliceosome associated protein homolog	Spliceosomal Bact complex	N.S.
CWC27	CWC27 spliceosome associated cyclophilin	Spliceosomal Bact complex	N.S.
DHX16	DEAH-box helicase 16	Spliceosomal Bact complex	N.S.
EFTUD2	elongation factor Tu GTP binding domain containing 2	Spliceosomal Bact complex	N.S.
GPATCH1	G-patch domain containing 1	Spliceosomal Bact complex	N.S.
GPKOW	G-patch domain and KOW motifs	Spliceosomal Bact complex	N.S.
PPIL2	peptidylprolyl isomerase like 2	Spliceosomal Bact complex	N.S.

Major splicoesome proteins (continue)

PRCC	proline rich mitotic checkpoint control factor	Spliceosomal Bact complex	N.S.
PRPF8	pre-mRNA processing factor 8	Spliceosomal Bact complex	N.S.
RNF113A	ring finger protein 113A	Spliceosomal Bact complex	N.S.
SAP18	Sin3A associated protein 18	Spliceosomal Bact complex	N.S.
SNRNP200	small nuclear ribonucleoprotein U5 subunit 200	Spliceosomal Bact complex	N.S.
SNRNP40	small nuclear ribonucleoprotein U5 subunit 40	Spliceosomal Bact complex	N.S.
ZNF830	zinc finger protein 830	Spliceosomal Bact complex	N.S.
CACTIN	cactin, spliceosome C complex subunit	Spliceosomal C complex	N.S.
CDK10	cyclin dependent kinase 10	Spliceosomal C complex	N.S.
DDX41	DEAD-box helicase 41	Spliceosomal C complex	N.S.
DHX35	DEAH-box helicase 35	Spliceosomal C complex	N.S.
DHX8	DEAH-box helicase 8	Spliceosomal C complex	N.S.
FAM32A	family with sequence similarity 32 member A	Spliceosomal C complex	N.S.
FAM50A	family with sequence similarity 50 member A	Spliceosomal C complex	N.S.
FRA10AC1	FRA10A associated CGG repeat 1	Spliceosomal C complex	-0.864965
HNRNPC	heterogeneous nuclear ribonucleoprotein C	Spliceosomal C complex	N.S.
HSPA8	heat shock protein family A (Hsp70) member 8	Spliceosomal C complex	N.S.
LENG1	leukocyte receptor cluster member 1	Spliceosomal C complex	N.S.
NOSIP	nitric oxide synthase interacting protein	Spliceosomal C complex	0.838361
PPIG	peptidylprolyl isomerase G	Spliceosomal C complex	N.S.
PPIL3	peptidylprolyl isomerase like 3	Spliceosomal C complex	N.S.
PPWD1	peptidylprolyl isomerase domain and WD repeat containing 1	Spliceosomal C complex	N.S.
PRPF18	pre-mRNA processing factor 18	Spliceosomal C complex	N.S.
SDE2	SDE2 telomere maintenance homolog	Spliceosomal C complex	N.S.
SLU7	SLU7 homolog, splicing factor	Spliceosomal C complex	N.S.
SRRM2	serine/arginine repetitive matrix 2	Spliceosomal C complex	N.S.
STEEP1	STING1 ER exit protein 1	Spliceosomal C complex	N.S.
SYF2	SYF2 pre-mRNA splicing factor	Spliceosomal C complex	N.S.
WDR83	WD repeat domain 83	Spliceosomal C complex	N.S.
DDX39B	DExD-box helicase 39B	Spliceosomal E complex	N.S.
BUD13	BUD13 homolog	Spliceosomal P complex	N.S.
C9orf78	chromosome 9 open reading frame 78	Spliceosomal P complex	N.S.
ESS2	ess-2 splicing factor homolog	Spliceosomal P complex	N.S.
SNIP1	Smad nuclear interacting protein 1	Spliceosomal P complex	N.S.
SART1	spliceosome associated factor 1, recruiter of U4/U6.U5 tri-snRNP	tri-snRP complex	N.S.
USP39	ubiquitin specific peptidase 39	tri-snRP complex	N.S.
SNRNP70	small nuclear ribonucleoprotein U1 subunit 70	U1 small nuclear ribonucleoprotein	N.S.
SNRPA	small nuclear ribonucleoprotein polypeptide A	U1 small nuclear ribonucleoprotein	N.S.
SNRPC	small nuclear ribonucleoprotein polypeptide C	U1 small nuclear ribonucleoprotein	N.S.
SNRPA1	small nuclear ribonucleoprotein polypeptide A'	U2 small nuclear ribonucleoprotein	N.S.
SNRPB2	small nuclear ribonucleoprotein polypeptide B2	U2 small nuclear ribonucleoprotein	N.S.
PPIH	peptidylprolyl isomerase H	U4/U6 small nuclear ribonucleoprotein	N.S.
PRPF3	pre-mRNA processing factor 3	U4/U6 small nuclear ribonucleoprotein	N.S.
PRPF31	pre-mRNA processing factor 31	U4/U6 small nuclear ribonucleoprotein	N.S.
PRPF4	pre-mRNA processing factor 4	U4/U6 small nuclear ribonucleoprotein	N.S.
SNU13	small nuclear ribonucleoprotein 13	U4/U6 small nuclear ribonucleoprotein	-0.815716
CD2BP2	CD2 cytoplasmic tail binding protein 2	U5 small nuclear ribonucleoprotein	N.S.
DDX23	DEAD-box helicase 23	U5 small nuclear ribonucleoprotein	N.S.
PRPF6	pre-mRNA processing factor 6	U5 small nuclear ribonucleoprotein	N.S.
TXNL4A	thioredoxin like 4A	U5 small nuclear ribonucleoprotein	N.S.

Minor splicoesome proteins

Symbol	Name	Group name	Log2 Fold Change (AGO123-/- / WT)	Overlaped with Major Spliceosome
PDCD7	programmed cell death 7	U11/U12 di-snRNP	N.S.	NO
RNPC3	RNA binding region (RNP1, RRM) containing 3	U11/U12 di-snRNP	N.S.	NO
SNRNP25	small nuclear ribonucleoprotein U11/U12 subunit 25	U11/U12 di-snRNP	N.S.	NO
SNRNP35	small nuclear ribonucleoprotein U11/U12 subunit 35	U11/U12 di-snRNP	N.S.	NO
SNRNP48	small nuclear ribonucleoprotein U11/U12 subunit 48	U11/U12 di-snRNP	N.S.	NO
YBX1	Y-box binding protein 1	U11/U12 di-snRNP	N.S.	NO
ZCRB1	zinc finger CCHC-type and RNA binding motif containing 1	U11/U12 di-snRNP	N.S.	NO
ZMAT5	zinc finger matrin-type 5	U11/U12 di-snRNP	N.S.	NO
ZRSR2	zinc finger CCCH-type, RNA binding motif and serine/arginine rich 2	U11/U12 di-snRNP	N.S.	NO
DHX15	DEAH-box helicase 15	U11/U12 di-snRNP	N.S.	YES
PHF5A	PHD finger protein 5A	SF3b complex	N.S.	YES
SF3B1	splicing factor 3b subunit 1	SF3b complex	N.S.	YES
SF3B2	splicing factor 3b subunit 2	SF3b complex	N.S.	YES
SF3B3	splicing factor 3b subunit 3	SF3b complex	N.S.	YES
SF3B4	splicing factor 3b subunit 4	SF3b complex	N.S.	YES
SF3B5	splicing factor 3b subunit 5	SF3b complex	N.S.	YES
SF3B6	splicing factor 3b subunit 6	SF3b complex	N.S.	YES
SNRPB	small nuclear ribonucleoprotein polypeptides B and B1	Sm spliceosomal proteins	N.S.	YES
SNRPD1	small nuclear ribonucleoprotein D1 polypeptide	Sm spliceosomal proteins	N.S.	YES
SNRPD2	small nuclear ribonucleoprotein D2 polypeptide	Sm spliceosomal proteins	-0.920	YES
SNRPD3	small nuclear ribonucleoprotein D3 polypeptide	Sm spliceosomal proteins	N.S.	YES
SNRPE	small nuclear ribonucleoprotein polypeptide E	Sm spliceosomal proteins	-0.744	YES
SNRPF	small nuclear ribonucleoprotein polypeptide F	Sm spliceosomal proteins	-0.752	YES
SNRPG	small nuclear ribonucleoprotein polypeptide G	Sm spliceosomal proteins	N.S.	YES
SNRPN	small nuclear ribonucleoprotein polypeptide N	Sm spliceosomal proteins	N.S.	YES
CD2BP2	CD2 cytoplasmic tail binding protein 2	U5 small nuclear ribonucleoprotein	N.S.	YES
DDX23	DEAD-box helicase 23	U5 small nuclear ribonucleoprotein	N.S.	YES
EFTUD2	elongation factor Tu GTP binding domain containing 2	U5 small nuclear ribonucleoprotein	N.S.	YES
PRPF6	pre-mRNA processing factor 6	U5 small nuclear ribonucleoprotein	N.S.	YES
PRPF8	pre-mRNA processing factor 8	U5 small nuclear ribonucleoprotein	N.S.	YES
SNRNP200	small nuclear ribonucleoprotein U5 subunit 200	U5 small nuclear ribonucleoprotein	N.S.	YES
SNRNP40	small nuclear ribonucleoprotein U5 subunit 40	U5 small nuclear ribonucleoprotein	N.S.	YES
TXNL4A	thioredoxin like 4A	U5 small nuclear ribonucleoprotein	N.S.	YES

SR proteins

Symbol	Name	Group name	Log2 Fold Change (AGO123-/- / WT)
SRSF1	serine and arginine rich splicing factor 1	Serine and arginine rich splicing factors	N.S.
SRSF2	serine and arginine rich splicing factor 2	Serine and arginine rich splicing factors	N.S.
SRSF3	serine and arginine rich splicing factor 3	Serine and arginine rich splicing factors	N.S.
SRSF4	serine and arginine rich splicing factor 4	Serine and arginine rich splicing factors	N.S.
SRSF5	serine and arginine rich splicing factor 5	Serine and arginine rich splicing factors	N.S.
SRSF6	serine and arginine rich splicing factor 6	Serine and arginine rich splicing factors	N.S.
SRSF7	serine and arginine rich splicing factor 7	Serine and arginine rich splicing factors	N.S.
SRSF8	serine and arginine rich splicing factor 8	Serine and arginine rich splicing factors	N.S.
SRSF9	serine and arginine rich splicing factor 9	Serine and arginine rich splicing factors	N.S.
SRSF10	serine and arginine rich splicing factor 10	Serine and arginine rich splicing factors	N.S.
SRSF11	serine and arginine rich splicing factor 11	Serine and arginine rich splicing factors	N.S.
SRSF12	serine and arginine rich splicing factor 12	Serine and arginine rich splicing factors	N.S.

hnRNP proteins

Symbol	Name	Log2 Fold Change (AGO123-/- / WT)
HNRNPC	heterogeneous nuclear ribonucleoprotein C	N.S.
HNRNPD	heterogeneous nuclear ribonucleoprotein D	N.S.
HNRNPF	heterogeneous nuclear ribonucleoprotein F	N.S.
HNRNPK	heterogeneous nuclear ribonucleoprotein K	N.S.
HNRNPL	heterogeneous nuclear ribonucleoprotein L	N.S.
HNRNPM	heterogeneous nuclear ribonucleoprotein M	N.S.
HNRNPR	heterogeneous nuclear ribonucleoprotein R	N.S.
HNRNPU	heterogeneous nuclear ribonucleoprotein U	N.S.
HNRNPA0	heterogeneous nuclear ribonucleoprotein A0	N.S.
HNRNPA1	heterogeneous nuclear ribonucleoprotein A1	N.S.
HNRNPA3	heterogeneous nuclear ribonucleoprotein A3	N.S.
HNRNPAB	heterogeneous nuclear ribonucleoprotein A/B	N.S.
HNRNPDL	heterogeneous nuclear ribonucleoprotein D like	N.S.
HNRNPH1	heterogeneous nuclear ribonucleoprotein H1	N.S.
HNRNPH2	heterogeneous nuclear ribonucleoprotein H2	N.S.
HNRNPH3	heterogeneous nuclear ribonucleoprotein H3	N.S.
HNRNPLL	heterogeneous nuclear ribonucleoprotein L like	N.S.
HNRNPCL1	heterogeneous nuclear ribonucleoprotein C like 1	N.S.
HNRNPCL2	heterogeneous nuclear ribonucleoprotein C like 2	N.S.
HNRNPCL3	heterogeneous nuclear ribonucleoprotein C like 3	N.S.
HNRNPCL4	heterogeneous nuclear ribonucleoprotein C like 4	N.S.
HNRNPUL1	heterogeneous nuclear ribonucleoprotein U like 1	1.249
HNRNPUL2	heterogeneous nuclear ribonucleoprotein U like 2	N.S.
HNRNPA1L2	heterogeneous nuclear ribonucleoprotein A1 like 2	N.S.
HNRNPA2B1	heterogeneous nuclear ribonucleoprotein A2/B1	N.S.

Other splicing associated proteins

Symbol	Name	Log2_Fold Change (AGO123-/- / WT)
AAR2	AAR2 splicing factor	N.S.
SFSWAP	splicing factor SWAP	N.S.
YJU2	YJU2 splicing factor homolog	N.S.
CENATAC	centrosomal AT-AC splicing factor	N.S.
ERH	ERH mRNA splicing and mitosis factor	N.S.
ESRP1	epithelial splicing regulatory protein 1	-3.36941
ESRP2	epithelial splicing regulatory protein 2	N.S.
KHSRP	KH-type splicing regulatory protein	N.S.
MBNL1	muscleblind like splicing regulator 1	N.S.
MBNL2	muscleblind like splicing regulator 2	N.S.
MBNL3	muscleblind like splicing regulator 3	N.S.
NOVA1	NOVA alternative splicing regulator 1	N.S.
NOVA2	NOVA alternative splicing regulator 2	N.S.
RP9	RP9 pre-mRNA splicing factor	N.S.
SFPQ	splicing factor proline and glutamine rich	N.S.
TSEN2	tRNA splicing endonuclease subunit 2	-1.33668
TSEN15	tRNA splicing endonuclease subunit 15	N.S.
TSEN34	tRNA splicing endonuclease subunit 34	N.S.
TSEN54	tRNA splicing endonuclease subunit 54	N.S.
YJU2B	YJU2 splicing factor homolog B	N.S.
NSRP1	nuclear speckle splicing regulatory protein 1	N.S.
LUC7L2	LUC7 like 2, pre-mRNA splicing factor	N.S.
LUC7L3	LUC7 like 3 pre-mRNA splicing factor	N.S.
KHDC4	KH domain containing 4, pre-mRNA splicing factor	N.S.
SREK1	splicing regulatory glutamic acid and lysine rich protein 1	N.S.
RBPMS	RNA binding protein, mRNA processing factor	1.75234
RBPMS2	RNA binding protein, mRNA processing factor 2	0.85117
SCAF4	SR-related CTD associated factor 4	N.S.
SUGP2	SURP and G-patch domain containing 2	N.S.
SCAF11	SR-related CTD associated factor 11	N.S.
SREK1IP1	SREK1 interacting protein 1	N.S.
DDX42	DEAD-box helicase 42	N.S.
TRPT1	tRNA phosphotransferase 1	N.S.
RSRC1	arginine and serine rich coiled-coil 1	0.586375
CLASRP	CLK4 associating serine/arginine rich protein	N.S.
PNISR	PNN interacting serine and arginine rich protein	N.S.
ARL6IP4	ADP ribosylation factor like GTPase 6 interacting protein 4	N.S.
RTCB	RNA 2',3'-cyclic phosphate and 5'-OH ligase	N.S.
AKAP17A	A-kinase anchoring protein 17A	N.S.
SRPK1	SRSF protein kinase 1	N.S.
SRPK2	SRSF protein kinase 2	N.S.
C1QBP	complement C1q binding protein	N.S.
TRA2A	transformer 2 alpha homolog	N.S.
TRA2B	transformer 2 beta homolog	N.S.

Down-regulated

Up-regulated

N.S. not significant
